# Supplementary material for: Maintenance of sarcomeric integrity in adult muscle cells crucially depends on Z-disc anchored titin
Source: Nat Commun. 2020 Sep 8;11:4479. doi: 10.1038/s41467-020-18131-2 (PMC7478974; doi:10.1038/s41467-020-18131-2)
Supplement: Supplementary file 1 — Supplementary Information [file 41467_2020_18131_MOESM1_ESM.pdf]

Supplement

Maintenance of sarcomeric integrity in adult muscle cells crucially depends on Z-disc anchored titin

Sandra Swist[1]*, Andreas Unger[2], Yong Li[2], Anja Vöge[1], Marion von Frieling-Salewsky[2], Åsa Skärlén[3], Nicola Cacciani[4], Thomas Braun[5], Lars Larsson[4], Wolfgang A. Linke[2]*

[1]Dept. of Systems Physiology, Ruhr University Bochum, D-44780 Bochum, Germany
[2]Institute of Physiology II, University of Munster, D-48149 Munster, Germany
[3]Department of Clinical Neuroscience, Clinical Neurophysiology, Karolinska Institute, SE-171 77 Stockholm, Sweden
[4]Department of Physiology and Pharmacology, Karolinska Institute, SE-171 77 Stockholm, Sweden
[5]Department of Cardiac Development and Remodeling, Max Planck Institute for Heart and Lung Research, D-61231 Bad Nauheim, Germany

*Corresponding authors:

Wolfgang A. Linke, Ph.D.
Institute of Physiology II, Univ. of Munster
Robert-Koch-Str. 27b, D-48149 Munster, Germany
Email: wlinke@uni-muenster.de

Sandra Swist, Ph.D.
Dept. of Systems Physiology, Ruhr Univ. Bochum
MA 02/146, D-44780 Bochum, Germany
Email: sandra.swist@ruhr-uni-bochum.de

Supplementary Methods and Data

Quality control of the generated Ttn^{tm1a} mice

Integrity of the inserted cassette and targeting of the Titin locus was validated in heterozygous Ttn$^{tm1a/+}$ mice by a set of PCR-based methods. We performed several short-range PCRs to confirm the presence of important features like loxP sites in the targeted mouse genome (Supplementary Fig. 1). Next, we performed a long-range PCR on the 3' prime end of the targeted locus with the primers P7 and P9 (Supplementary Fig. 1a, c). The resultant PCR product, sequenced by the LR primer, confirmed correct targeting of the locus and the presence of the downstream loxP site (Supplementary Fig. 1 a, c). For primers used, see Supplementary Table 1.

LacZ stainings

For whole mount LacZ stainings, tissues were fixed in a buffered glutaraldehyde solution (5 mM EGTA, 0.4 % glutaraldehyde, 2 mM $MgCl_2$ in 0.1 M phosphate buffer), washed in LacZ washing buffer (2 mM $MgCl_2$, 0.01 % sodium deoxycholate and 0.02 % Nonidet® P40 in 0.1 M phosphate buffer) and stained in LacZ staining solution (5 mM potassium ferricyanide, 0.1 % X-gal in LacZ washing buffer) until the desired color intensity was achieved. After staining, tissues were further fixed in 4 % paraformaldehyde at 4 °C overnight and then photographed using a Leica IC80 HD camera attached to a Leica Mz125 stereo microscope.

Cryosections were stained after post-fixation in buffered glutaraldehyde solution and stained with the above-mentioned staining solution, washed in phosphate buffer, mounted in Mowiol and photographed using a Nikon DS-Fi2/DS-U3 camera attached to a Leica DMRBE research microscope.

Antibodies

Antibodies against murine TTN isoforms Novex-3 and Cronos were custom-made. Peptide synthesis, coupling to the Keyhole Limpet Hemocyanin (KLH) carrier, antibody production and purification were conducted by Kaneka Eurogentec S.A. (Seraing, Belgium). For Novex-3 antibody production, rabbits were immunized with the peptide C+PNE AIE PKD NEM PPS. For the generation of the Cronos antibody, rabbits or guinea pigs were immunized with the peptide SNT RHF VSS RKA+C. The Novex-3 antibody was validated on WT and TTN KO tissue, while the Cronos antibody was validated by a peptide competition protocol. During this procedure, antibodies were pre-incubated with a saturating concentration of the antigenic peptide used for immunization for 1 h a 4 °C, prior to application onto western blots. The monoclonal antibodies against Lamp-2 (ABL-93; by J. T. August), myosin heavy chain (MF 20; by D. A. Fischman) and myosin heavy chain type IIB (BF-F3; by S. Schiaffino) were obtained from the Developmental Studies Hybridoma Bank developed under the auspices of the NICHD and maintained by The University of lowa, Department of Biology, lowa City, 1A52242, USA. Further information about antibodies and dilutions is provided in Supplementary Table 2.

Generation of TTN knockout mice in a conditional design

Knockout-first heterozygous Ttn$^{tm1a/+}$ mice were viable, fertile, and had a normal life span. We validated the integrity of the inserted cassette and confirmed targeting of *Ttn* exons 4-6 by PCR (Supplementary Fig. 1). In this conditional design, the initial, unmodified *tm1a* allele generates a null allele by splicing *Ttn* exon 3 to the *lacZ* trapping element present in the targeting cassette, involving a strong splice acceptor (Supplementary Fig. 2a). The resultant fusion protein comprises the TTN N-terminus encoded by exons 1-3 and β-galactosidase (Supplementary Fig. 2a), after which the translation is terminated. As

expected[1-3], inbreeding $Ttn^{tm1a/+}$ mice to homozygosity was unsuccessful (38 pups, 9 litters). Therefore, we screened for homozygous animals during embryonic development by using PCR on yolk-sac DNA (Supplementary Fig. 2b). At embryonic day (E)9.5, we observed embryos in a Mendelian distribution, whereas no homozygous mutant embryo survived beyond E11.5. At E10.5, we found growth-retarded embryos with enlarged pericardium (Supplementary Fig. 2c) or resorption bodies. Thus, titin-deficient embryos die around E10.

RT-PCR analyses revealed loss of the full-length *Ttn* message in homozygous mutant E9.5 embryos, using primers to exons 1 and 8 (Supplementary Fig. 2d). *Ttn* mRNA was also detected in mouse lungs, likely because cardiac muscle fibers extend from the left atrium along the pulmonary veins as far as the venous radicles[4]. Quantitative PCR (qPCR) analyses (Supplementary Fig. 2e) revealed downregulation of *Ttn* exons 3-4 by 98±2% (difference between mean±SEM), relative to canonical *Ttn* exons 1-2. Expression of *Ttn* exons 20-21 was reduced by 74±4%, while Cronos mRNA expression was unaltered in mutant embryos. On protein gels, no full-length TTN was detected in MUT animals, whereas a signal was still present at the position expected for Cronos (Supplementary Fig. 2f). Titin expression was examined in different tissues of heterozygous $Ttn^{+/tm1a}$ mice by using the LacZ cassette spliced to *Ttn* exon 3 and reading out the lacZ staining (Supplementary Fig. 3). Findings were consistent with the RT-PCR data for adult WT tissues (Supplementary Fig. 2d). Thus, the Ttn^{tm1a} mouse line is suitable for reporting TTN expression from the canonical promoter. By ultrastructural analyses of E9.5 hearts, it was confirmed that homozygous MUT hearts failed to develop aligned sarcomeres, although Z-disc-like condensations and thick filaments were present (Supplementary Fig. 2g).

Supplementary Tables

Supplementary Table 1: Primers used in this study.

Name	Sequence from 5' to 3'	Binds to	Accession No.	Usage
**LR Primer**	ACTGATGGCGAGCTCAGACC	*synthetic loxP region*	JN959336	cassette integrity
**P2/5' Uni-versal (LAR3)**	CACAACGGGTTCTTCTGTTAGTCC	*mouse engrailed-2 intron*	JN959336	cassette integrity
**P5/lacZ_fwd1**	CCGACGGCACGCTGATTGAAG	lacZ	JN959336	cassette integrity
**P6/lacZ_rev1**	ATACTGCACCGGGCGGGAAGGAT	lacZ	JN959336	cassette integrity
**P7/Ttn 3'LR fwd**	CCCACTCACCACGGCTTCTGC	*Ttn intron 5*	JN959336/ BN001114	cassette integrity
**P8/Ttn 3'armR**	TGGGAAAGCCCCCTACCTCCT	*Ttn intron 6*	JN959336/ BN001114	cassette integrity
**P1/Ttn_In3_F**	GCACTCACCTTACCAGCCACGG	Ttn Intron3	BN001114	cassette integrity/ genotyping
**P3/3' Uni-versal (RAF5)**	CACACCTCCCCCTGAACCTGAAAC	SV40 poly-adenylation site	JN959336	cassette integrity/ genotyping
**P4/Ttn_In3_R**	AGGCAGCAGGAGCAGAAGGCT	Ttn Intron3	BN001114	cassette integrity/ genotyping
**P9/GR4**	CATATCAACAGCAGCAACGACAGTCGCAAC	*Ttn exon 8*	BN001114	cassette integrity/ RT-PCR
**Cre-1**	GTTCGCAAGAACCTGATGGACA	Cre cds	2777477	genotyping
**Cre-2**	CTAGAGCCTGTTTTGCACGTT	Cre cds	2777477	genotyping
**Flp-F**	CTGGAGGATAACTTGTTTATTGC	FLP cloning vector	CVU46493	genotyping
**Flp-R**	CTAATGTTGTGGGAAATTGGAGC	Flp cds	CVU46493	genotyping
**oIMR8744**	CAAATGTTGCTTGTCTGGTG	Acta1-TetO-cre internal positive control		genotyping
**oIMR8745**	GTCAGTCGAGTGCACAGTTT	Acta1-TetO-cre internal positive control		genotyping
**oIMR8746**	CGCTGTGGGGCATTTTACTTTAG	Acta1-TetO-cre		genotyping
**oIMR8747**	CATGTCCAGATCGAAATCGTC	Acta1-TetO-cre		genotyping
**Rosa-FA**	AAAGTCGCTCTGAGTTGTTAT	Rosa Locus	14910	genotyping
**Rosa-RF**	GGAGCGGGAGAAATGGATATG	Rosa Locus	14910	genotyping
**RosaSplicAC**	CATCAAGGAAACCCTGGACTACTG	Splice Acceptor Rosa26_LacZ		genotyping
**TtnP002_F**	TTTCAAAGCCGGTGGTCCAG	*Ttn exon 20*	BN001114	probe amplification for in situ hybridizn.

Name	Sequence from 5' to 3'	Binds to	Accession No.	Usage
**TtnP002_R**	CACGTACAGCTTCCCAGAGC	*Ttn exon 24*	BN001114	probe amplification for in situ hybridizn.
**qACTA1_F**	ATCCAGGCGGTGCTGTCCCT	*Acta1 exon 4*	NM_001272041	qPCR
**qACTA1_R**	ATGATGGCGTGTGGCAGGGC	*Acta1 exon 5*	NM_001272041	qPCR
**qCronos_F**	ACAAGGCATTTTGTTTCCTCTCGCA	*Cronos 5'UTR*	BN001114	qPCR
**qmHPRT1**	GATTCAACTTGCGCTCATCTTA	Hprt exon 9	NM_013556	qPCR
**qmHPRT2**	GTTGGATACAGGCCAGACTTTGTTG	Hprt exon 7/8	NM_013556	qPCR
**qMhy4_F**	GTCCTTCCTCAAACCCTTAAAGT	*Myh4 exon 1*	NM_010855	qPCR
**qMyh4_R**	CTATTGGTGGCAGCTCAGGG	*Myh4 exon 3*	NM_010855	qPCR
**qTtnP001_F**	ACTTGGGCTTGGTCCCACCCT	*Ttn exon 1*	BN001114	qPCR
**qTtnP002_R**	CGGCTGCGTAAACATCGGTGC	*Ttn exon* 2	BN001114	qPCR
**qTtnP003_F**	TCCCCGCCGTGACTAAAGCC	*Ttn exon 3*	BN001114	qPCR
**qTtnP004_R**	GGTGGTGCTGTCTCGGCTGTC	*Ttn exon 4*	BN001114	qPCR
**qTtnP020_F**	CGGCAACCCAAAGCCCCACG	*Ttn exon 20*	BN001114	qPCR
**qTtnP021_R**	CCAGTCTGCATTCCCCAGTCTGC	*Ttn exon 21*	BN001114	qPCR
**qTtnP239_F**	GCTGCCAATGTCAAATCTAGTGCCCA	*Ttn exon 239*	BN001114	qPCR
**qTtnP240_R**	AAGGTGGCCGTTTCCCCTGC	*Ttn exon 240*	BN001114	qPCR
**TtnP004_R**	AGTCAAAGGTGGCCGTTTCC	*Ttn exon 240*	BN001114	qPCR (detection of Cronos in comb-ination with primer "qCronos_F")
**musHPRT_F**	CACAGGACTAGAACACCTGC	Hprt exon 9	NM_013556	RT-PCR
**musHPRT_R**	GCTGGTGAAAAGGACCTCT	Hprt exon 7	NM_013556	RT-PCR
**TtnP009**	GAGGGTTAGAGGCTCACCGA	*Ttn exon 1*	BN001114	RT-PCR
**Ttn001**	GGAGGGTAGTACCGCAACCT	*Ttn exon* 2	BN001114	RT-PCR

Supplementary Table 2: Primary and secondary antibodies used in this study. Antibody dilutions for the different procedures are given. WB: western blot, IHC, immunohistochemistry; HRP, horseradish peroxidase; IgG, Immunoglobulin G; IEM, immunoelectron microscopy.

		Antibody dilution			
**Antibody**	**Company, Catalog No.**	**Western blot**	**IHC**	**IEM**	**Validation**
**Actinin alpha 2 (EA53)**	Sigma, A7811	1:2.500	1:100	1:100	WB: Manufacturer's website; Detection of expected band in rat leg muscle, IHC: human cardiomyocytes (Gao et al., 1997; J Biol Chem, 272, 19401-7).
**Alpha B Crystallin**	Abcam, ab76467	1:1.000			WB: Manufacturer's website; Detection of expected band in rat eyeball lysate.
**ANKRD1**	Sigma, HPA038736	1:1.000			WB: Detection of expected bands in murine skeletal muscles in the present study.
**ANKRD1**	Myomedix, ANKRYD1-1	1:1.000			WB: Validation in heart extracts from WT und ANKRD1 knockout mice (Lange et al., 2016; Nat Commun, 7, 12120).
**ANKRD2**	Sigma, AV42559	1:1.000			WB: Detection of expected 42 kDa band in murine skeletal muscle extract in the present study.
**Calpain-1**	Santa Cruz, sc-7530	1:200			WB: Manufacturer's website; Detection of expected band in different cell lines.
**Calpain-3**	Novocastra (Leica), NCL-CALP-12A12	1:500			WB: Validated by detection of expected bands in skeletal muscles from different animals (Anderson et al., 1998, Am J Pathol, 153, 1169-79).
**CSRP3 (MLP)**	Gift of R. Knöll	1:3.000			Reference: Knöll et al., 2002; Cell, 111, 943-955. (WB: Validated by detection of expected bands in muscle lysates)
**Cy™3 AffiniPure Goat Anti-Rabbit IgG**	Jackson ImmunoResearch, 111-165-003		1:100		IHC: Manufacturer's website
**FHL1**	Abcam, ab49241	1:1.000			WB: Manufacturer's website ; Detection of expected band in fetal muscle lysates, detection of expected band in murine pulmonary arterial smooth muscle cells (Veith et al., 2012; Am J Pathol, 181, 1621-33).
**FHL2**	Abcam, ab12327	1:1.000			WB: Validation in murine WT and FHL2-null fibroblasts (Labalette et al., 2008; J Biol Chem, 283, 15201-8).
**GAPDH**	Cell Signaling, 2118				WB: Manufacturer's website; Detection of expected band in various cell lines.
**HSC70**	Abcam, ab2788	1:1.000			WB: Manufacturer's website; Detection of expected band in murine NIH3T3 cells and different human cell lines
**HSP70**	Abcam, ab2787	1:1.000			WB: Manufacturer's website; Detection of expected band in different human cell lines; detection of expected band in the murine cell line 3T3-L1 (Lemecha et al., 2018; Sci Rep, 8, 15096).
**HSP90(β)**	Abcam, ab51145	1:1.000			WB: Manufacturer's website; Validated on HeLa cell extracts by peptide competition assay

Antibody	Company, Catalog No.	Antibody dilution<br>Western blot	IHC	IEM	Validation
**Lamp-2**	Developmental Studies Hybridoma Bank, ABL-93	1:100			WB: Liver and kidney of Lamp-2 WT and knockout mice (Tanaka et al., 2000; Nature, 406, 902-906)
**Mouse IgG F(ab')2 Antibody Fluorescein Conjugated**	Rockland, 610-1204		1:300		IHC: Manufacturer's website
**Myomesin 1**	clone BB78, gift of W. Obermann, Bochum	1:1.000			WB: Obermann et al., 1995; Eur J Biochem, 233, 110-5. (Validated by detection of expected bands in muscle lysates and cells.)
**Myosin, heavy chain**	Developmental Studies Hybridoma Bank, MF 20		1:40		IHC: Validated in Bader et al., 1982; J Biol Chem, 95, 763-70.
**Myosin**, heavy chain **type IIB**	Developmental Studies Hybridoma Bank, BF-F3	1:2.000			WB: Validated using mouse masseter muscle (Widmer et al., 2002; J Dent Res; 81:33-8).
**Peroxidase-conjugated anti-goat IgG**	Merck, AP106P	1:5.000			WB: Manufacturer's website
**Peroxidase-conjugated goat anti-Guinea Pig IgG**	ImmoReagents, GtxGp-003-DHRPX	1:5.000			WB: Manufacturer's website
**Peroxidase-conjugated goat anti-Mouse IgG**	Dianova/Jackson ImmunoResearch Laboratories, 115-035-146	1:5.000			WB: Manufacturer's website
**Peroxidase-conjugated goat anti-Rabbit IgG**	Acris, R1364HRP	1:10.000			WB: Manufacturer's website
**Peroxidase-conjugated Goat IgG anti-Rat IgG**	Dianova/Jackson ImmunoResearch Laboratories, 112-035-003	1:5.000			WB: Manufacturer's website
**Smyd1**	Sigma, SAB2108345	1:1.000			WB: Manufacturer's website; Detection of expected band in HeLa cell lysates.
**SQSTM1**	Progen, GP62	1:1.000			WB: Validation in knockout mice (Antonucci et al., 2015; Proc Natl Acad Sci USA, 112, E6166-74).
**Telethonin (N-20)**	Santa Cruz Biotechnology, sc-8725	1:200			WB: 1) Manufacturer's website; Detection of expected band in mouse heart extracts; 2) validated on bacterially expressed T-Cap (Kontrogianni-Konstantopoulos & Bloch, 2003; J Biol Chem, 278, 3985-91).
**TTN gup-*a*-mCronos**	custom-made, Kaneka Eurogentec S.A.	1:4.000			WB & IHC: Validated in this study (in addition to Cronos, the antibody detects an unspecific band of ~0.75 MDa).

Antibody	Company, Catalog No.	Antibody dilution Western blot	IHC	IEM	Validation
**TTN Kinase**	Myomedix, TTN-8		1:200	1:200	WB: Validation in knockout mice (Gotthardt et al., 2003; J Biol Chem, 278, 6059-65); IHC: validation in knockout mice in the present study.
**TTN MIR**	custom-made, Kaneka Eurogentec S.A.			1:200	IEM: Validation in WT and knockout mice in the present study.
**TTN Novex-3**	custom-made, Kaneka Eurogentec S.A.	1:1.000	1:100		WB, IHC: Validated in WT and knockout mice in the present study.
**TTN rabbit-*a*-mCronos**	custom-made, Kaneka Eurogentec S.A.	1:4.000	1:100		WB: Validated in this study (in addition to Cronos, the antibody detects an unspecific band of ~0.75 MDa).
**TTN Z/I (2080)**	custom-made, Kaneka Eurogentec S.A.	1:1.0000	1:200	1:100	WB, IHC: Krysiak et al., 2018; Nat Commun, 9, 262. Detection of expected band in cardiac muscle cells.
**TTN Z/I (T12)**	gift of D. Fürst, Bonn			1:100	WB, IHC, IEM: Furst et al., 1988; J Cell Biol, 106, 1563-72. Detection of expected band in cardiac muscle cells.
**Ub (P4D1)**	Santa Cruz Biotechnology, sc-8017	1:1.000			WB: Manufacturer’s website; Validation in non-transfected and transfected 293T cells; detection of multiple ubiquitinated bands.

Supplementary Figures

Supplementary Figure 1

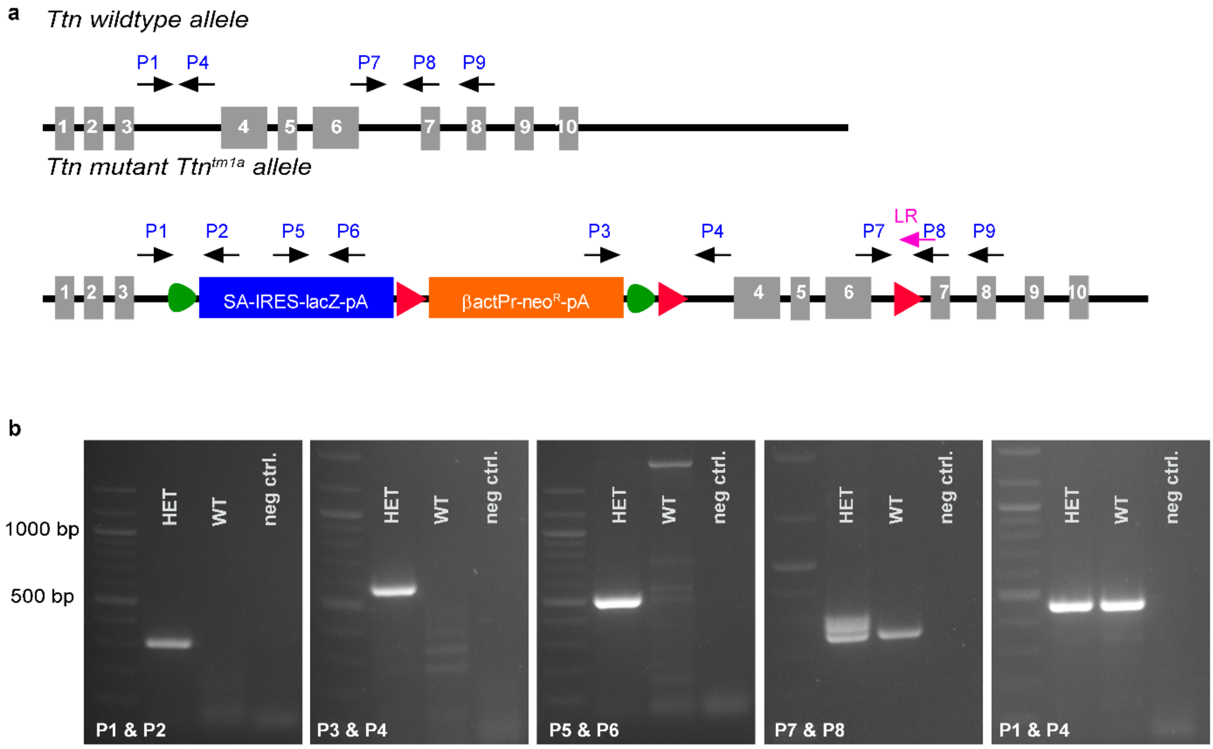

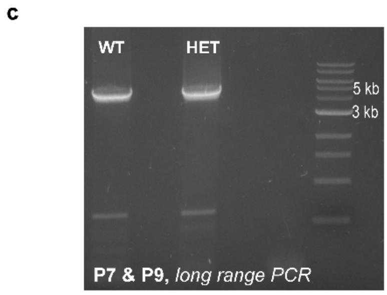

d

Primer pairs	WT allele	MUT allele
P1 & P2	no amplicon	318 bp
P3 & P4	no amplicon	575 bp
P5 & P6	no amplicon	506 bp
P1 & P4	451 bp	no amplicon
P7 & P8	751 bp	780 bp
P7 & P9	*5062 bp*	*5091 bp*

Supplementary Figure 1: Titin wildtype and Ttn^{tm1a}allele structure. a) Schematic of the titin wildtype and mutant knockout-first' allele (tm1a) containing a trapping/selection cassette inserted into intron 3 of the titin gene. This cassette is flanked by FRT sites (green triangles) and contains the LacZ trapping element (blue square) with the splice acceptor (SA) and an internal ribosomal entry site (IRES). LoxP sites are depicted as red triangles. The resistance gene neomycin (neoR) is driven by the human β-actin promoter (βactPr) and is followed by a simian virus terminator and polyadenylation signal (pA). Primers for the integrity check via PCR are indicated by black arrows. The LR primer (magenta) served as sequencing primer. **b)** Short-range PCR analyses of heterozygous Ttn$^{tm1a/+}$ (HET) and wildtype Ttn$^{+/+}$ (WT) alleles. NTC, non-template control. **c)** Long-range PCR analysis. The approximately 5 kb amplicons were further analyzed by Sanger sequencing using the LR primer (magenta color) depicted in a). **d)** Overview of the expected PCR results. The agarose gels shown are representative of 2 gels performed per condition.

Supplementary Figure 2

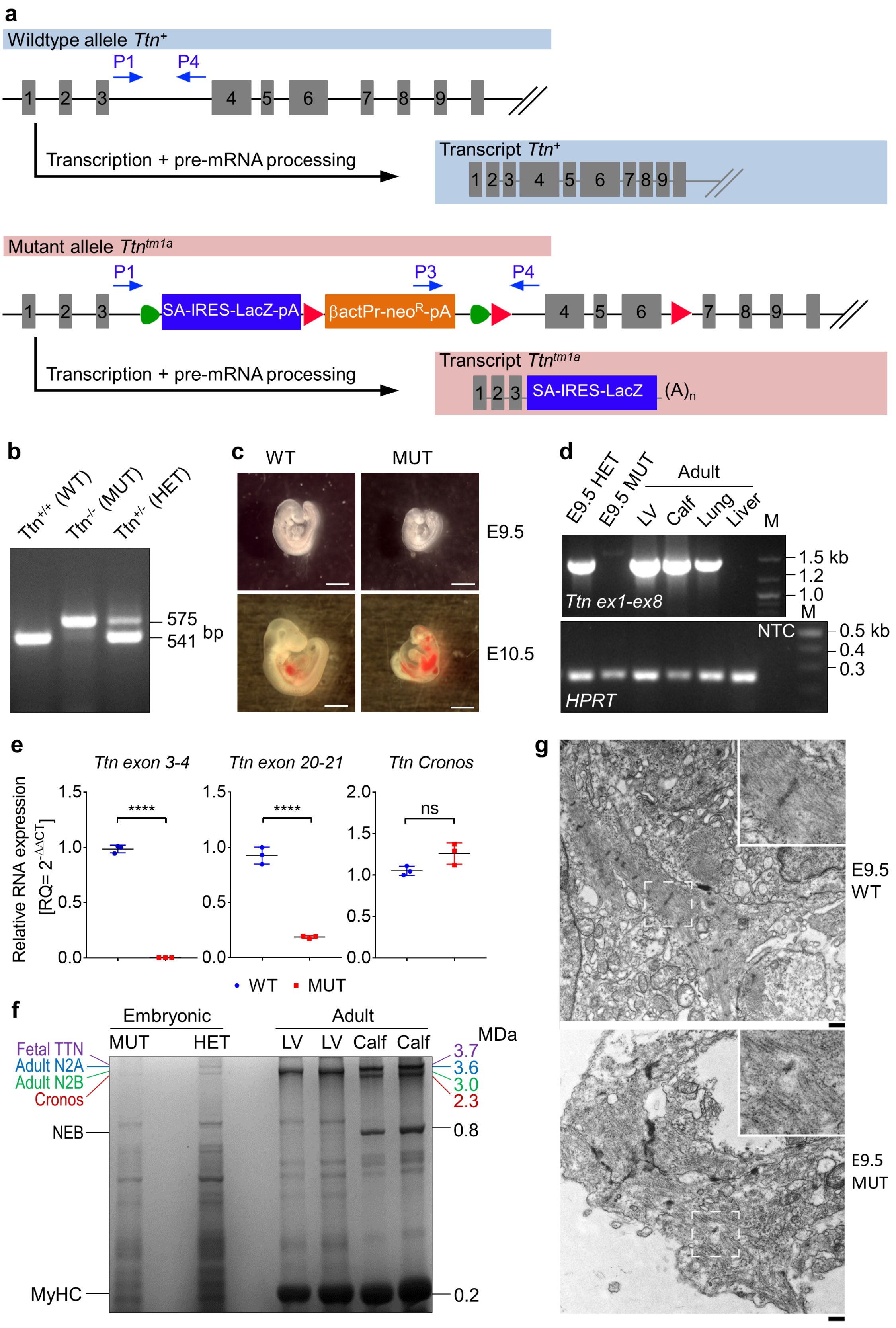

Supplementary Figure 2: The constitutive titin knockout Ttn^{tm1a} is embryonic lethal. a) Schematic of wildtype (WT) and mutant Ttn^{tm1a} alleles. Exons are shown as grey boxes, *LoxP* sites as red and *Frt* sites as green triangles. Blue arrows denote the positions of primers (P1, P3, P4) used for PCR genotyping. **b)** PCR-based genotyping; a representative agarose gel is shown (out of at least 4 similar gels performed). Products derived from WT and

homozygous (MUT) alleles were 451 bp and 575 bp; both alleles were present in heterozygous animals (HET). **c)** Images of WT and homozygous MUT embryos at two different days of embryonic development (E9.5, E10.5); images are representative of 15 images recorded per condition at stage 9.5 and 5 images recorded per condition at stage 10.5. Scale bars, 1 mm. **d)** RT-PCR analysis of *Ttn* RNA/cDNA from exons 1-8 (1.4 kb) revealing complete loss of the full-length *Ttn* message in homozygous mutant E9.5 embryos. Adult tissues are from WT C57Bl/6J mice. HPRT-PCRs served as endogenous control. LV, left ventricle; Calf, calf muscle; M, marker; NTC, non-template control. **e)** Results of qPCR analyses of Ttn^{tm1a} Knockout-first homozygous MUT and WT embryos at E9.5. Downregulation in MUT vs. WT was found for Ttn exons 3-4 (n=3 animals/group, p<0.0001) and 20-21 (n=3 animals/group, p<0.0001) but not for the Cronos Ttn transcript (n=3 animals/group, p=0.0607). Data (mean±SD) normalized to the expression level of Ttn exons 1-2; ****p<0.0001; unpaired, two-tailed Student's t-test. **f)** Coomassie-stained gel (one of two similar gels performed) showing TTN-protein expression in MUT and HET embryos at E9.5 (for each lane, 3 embryos were pooled). Adult left ventricle (LV) and adult skeletal muscle (Calf) served as size standards. **g)** Electron micrographs of E9.5 Ttn^{tm1a} WT and MUT hearts. Images are representative of 20 micrographs/genotype recorded. Scale bars, 1 µm. Insets, 2.5x magnified regions. Source data are provided as a Source Data file.

Supplementary Figure 3

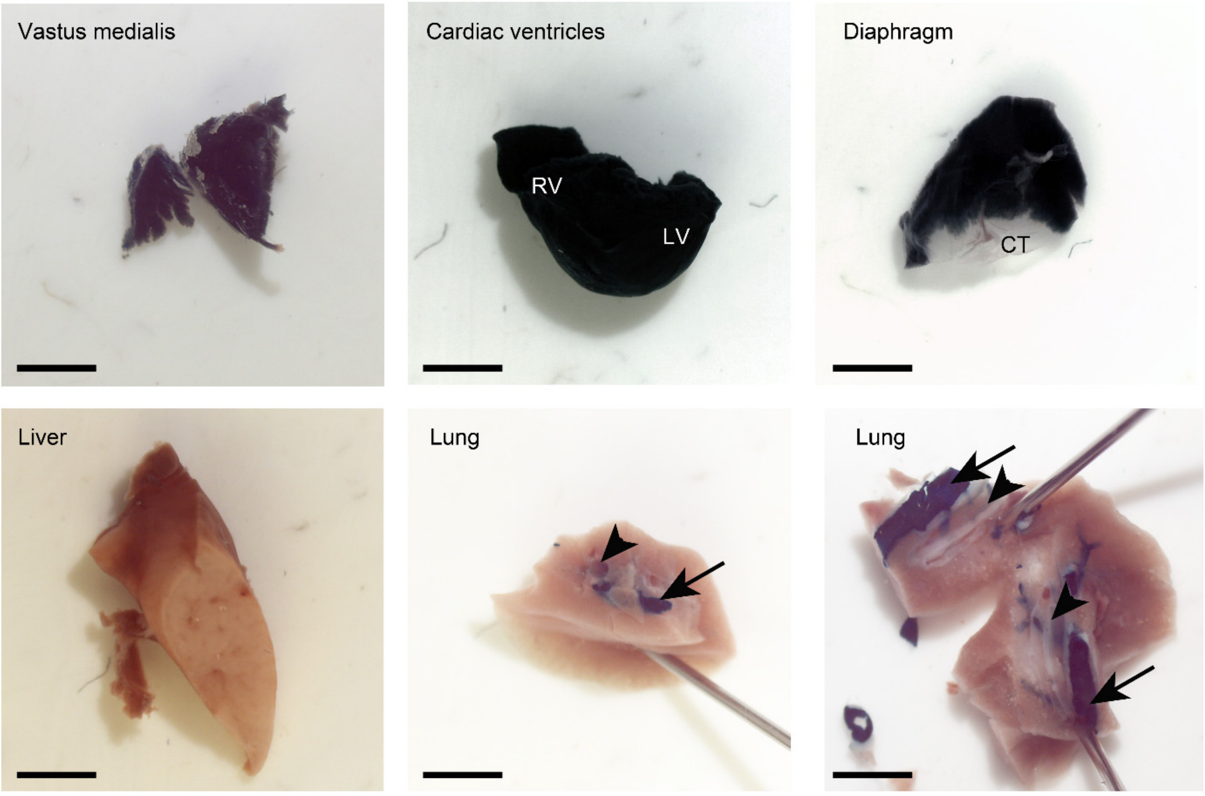

Supplementary Figure 3: LacZ staining of diverse heterozygous Ttn^{tm1a} tissues. Muscle cells stain blue after the staining procedure, while non-muscle tissue like liver and the parenchyma remain unstained. The lower left panel is a top view of the trimmed edge of a sagittal section of a liver lobe. Pieces of the lung are pinned to the underlay using small needles. The lung piece on the lower right panel was dissected along the big vessels to visualize the selective staining along the pulmonary vein (arrows), while the bronchus (arrowheads) remains unstained. LV, left ventricle; RV, right ventricle; CT, central tendon; Scale bars, 2 mm.

Supplementary Figure 4

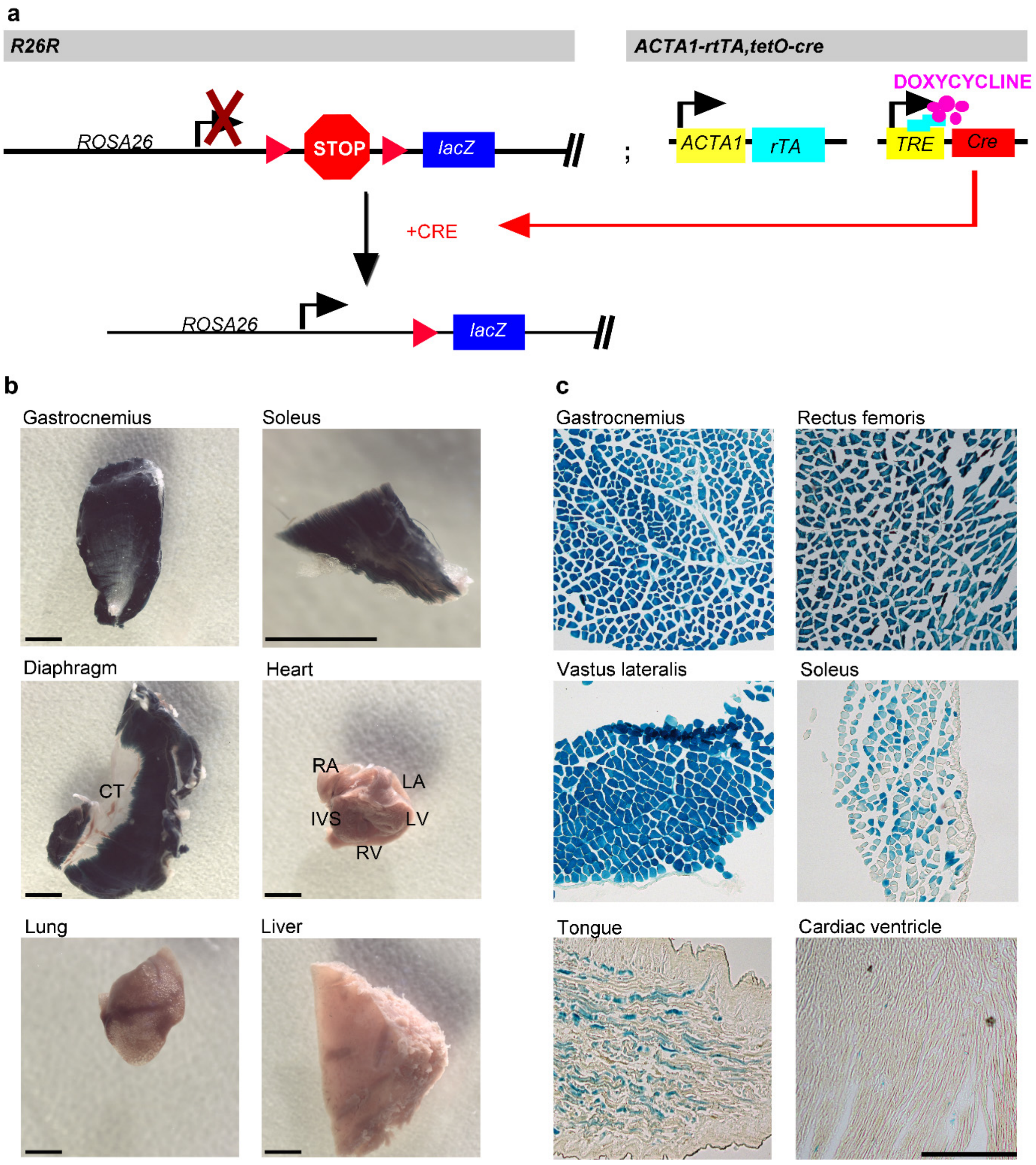

Supplementary Figure 4: CRE expression in the ACTA1-TA/tetO-cre line after 30 days of dox-treatment. a) Schematic demonstrating the use of the *lacZ* reporter for the detection of dox-induced Cre expression. The R26R recombinant locus harbors a loxP-flanked stop cassette preventing the expression of lacZ from the ubiquitously expressed Rosa26 promoter. In the hybrid line R26R/ACTA1-rtTA;tetO-cre, CRE is expressed following dox-treatment under the control of the human actin, alpha 1 skeletal muscle (*ACTA1*) promoter and CRE deletes the transcriptional stop, which leads to *lacZ* expression, **b)** LacZ-stained tissues dissected from R26R/ACTA1-rtTA;tetO-cre mice after 30 days of dox-treatment. Scale bars, 2 mm. RA, right atrium; LA, left atrium; IVS, interventricular septum; LV, left ventricle; RV, right ventricle. **c)** LacZ staining on cryosections of different tissues from the R26R/ACTA1-rtTA;tetO-cre hybrid line after 30 days of dox-treatment. Images are representative of 2-4 micrographs/muscle type recorded. Scale bar, 250 µm.

Supplementary Figure 5

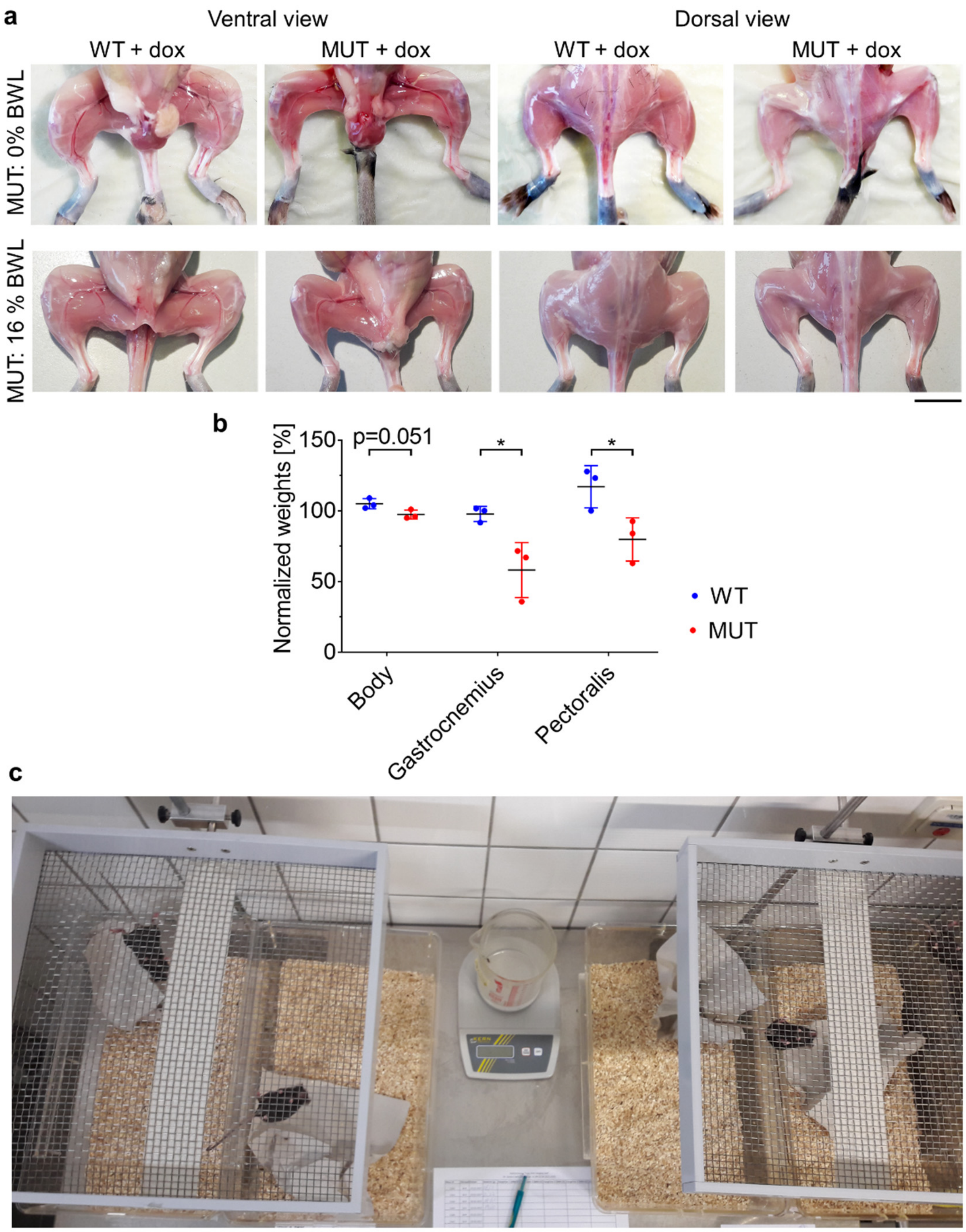

Supplementary Figure 5: Phenotyping of WT and MUT mice. a) Ventral and dorsal views of skinned WT and MUT Ttn^{tm1c};ACTA1-rtTA;tetO-cre hindlimbs from mice treated with doxycycline for 21 days (upper row) or 30 days (lower row), demonstrating the muscle atrophy in MUT. Scale bar, 1 cm; BWL, body weight loss. **b)** Relative body and muscle weights of WT and MUT after 21 days of doxycycline treatment. Body weight was normalized to the initial body weight before dox-treatment; muscle weights were indexed to tibia length. n=3 animals/group; *$p_{Gastrocnemius}$=0.0273; *$p_{Pectoralis}$=0.0392. Graph shows mean±SD. All p-values were calculated using two-tailed unpaired Student's t-test. **c)** Experimental setup of the four limb hanging test. Source data are provided as a Source Data file.

Supplementary Figure 6

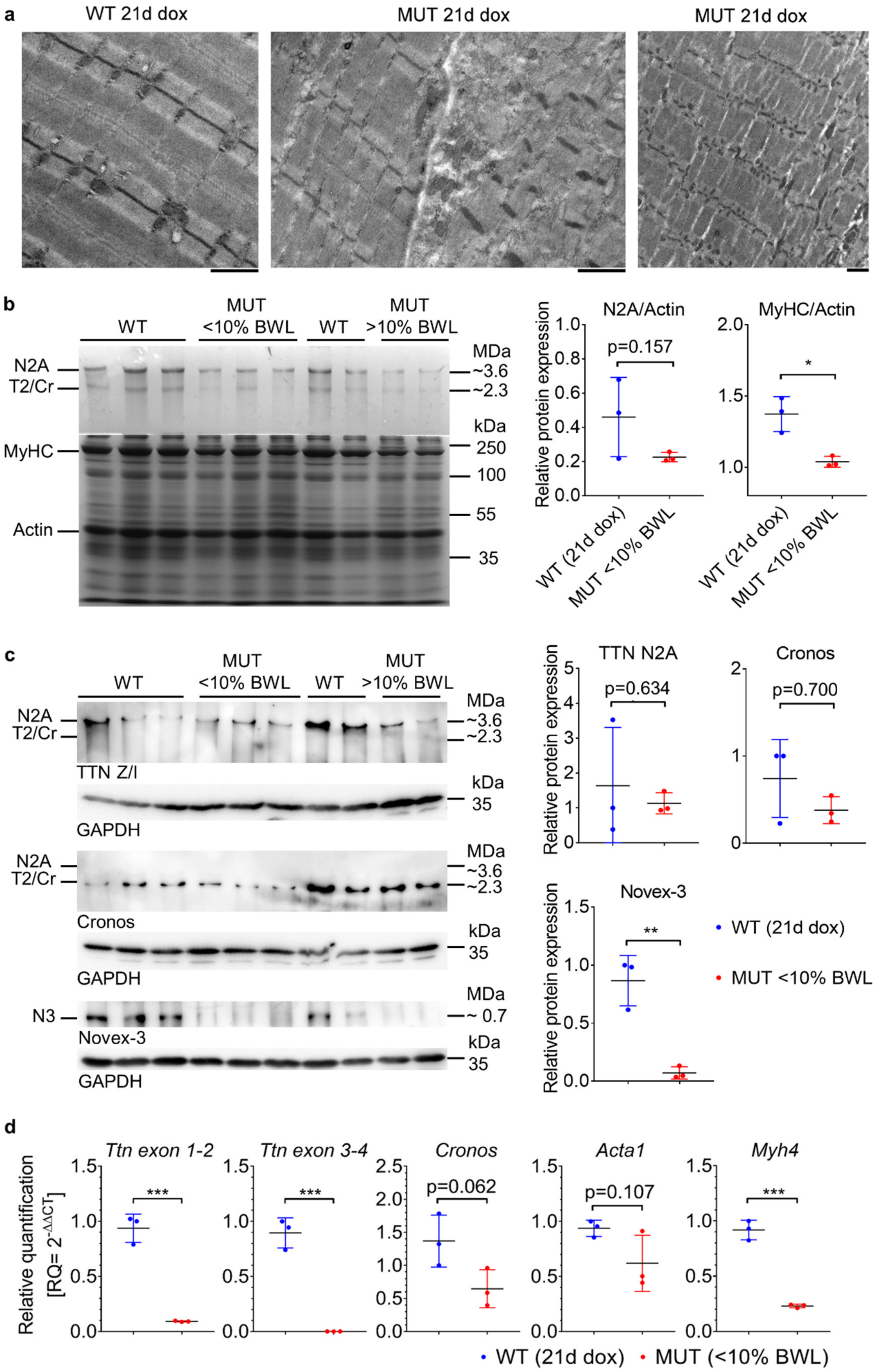

Supplementary Figure 6: Muscle ultrastructure, protein and transcript expression 21 days of dox-treatment. **a**) Electron micrographs of WT and MUT mouse gastrocnemius myocytes after dox-treatment for 21 days. Right

two panels show areas already affected by the TTN deletion, while in most cells the normal substructure was still maintained. Scale bars, 1 µm. Images are representative of at least 15 micrographs recorded per animal (n=2 animals/genotype). **b)** Coomassie-stained titin gel (2.5% polyacrylamide) stacked onto a 10% running gel and loaded with samples of WT (n=3) and MUT (n=3; <10% body weight loss (BWL)) Ttn^{tm1c};ACTA1-rtTA;tetO-cre pectoralis muscle after 21 days of dox-treatment. For comparision, we added WT (n=2) and MUT (n=2; 10-20% BWL) muscles after ~30 days of dox-treatment. Right panels show data from densitometric analysis at 21 days of dox-treatment, comparing TTN (N2A) and MyHC to actin levels (n=2-3; *p=0.0011). MyHC, myosin heavy chain; T2, titin proteolytic fragment; Cr, Cronos isoform. **c)** Western blots showing expression of the different TTN isoforms in MUT pectoralis muscles at earlier (<10% BWL) and later time-points (10-20% BWL) of dox-treatment, and the respective WT controls. GAPDH, loading control. N3, Novex-3 isoform. Right panels show data from densitometric analysis at 21 days of dox-treatment, indexed to GAPDH (n=3 mice/group); **$p_{Novex\text{-}3}$=0.0035; ns, non-significant. **d)** Relative mRNA expression of Ttn exon 1-2, exon 3-4, Cronos, alpha 1 skeletal muscle actin (*Acta1*), and *Myh4* (codes for MyHC-IIb) in gastrocnemius muscles after 21 days of dox-treatment, measured by real-time qPCR; n=3 mice/group. ***$p_{Ttn\ exon\ 1\text{-}2}$=0.0003; ***$p_{Ttn\ exon\ 3\text{-}4}$=0.0003; ***$p_{Myh4}$=0.0002. Graphs show mean±SD. All p-values were calculated using two-tailed unpaired Student's t-test. Source data are provided as a Source Data file.

Supplementary Figure 7

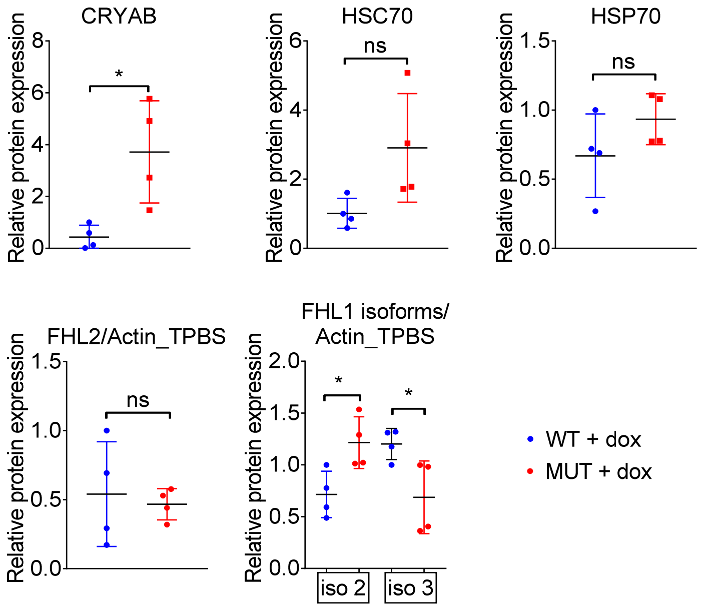

Supplementary Figure 7: Expression of titin-binding proteins and chaperones in MUT vs. WT skeletal muscles. Relative protein expression of specified proteins in murine WT and MUT pectoralis muscles after ~30 days of dox-treatment. Protein expression was either normalized to immunodetected GAPDH or to actin in the total protein blot stain (TPBS). FHL1 isoforms (iso) 3 and 2 were both detected by the same antibody. Individual data points and means±SD are shown; n=4 animals/group; *p<0.05; unpaired, two-tailed Student's t-test. ns, non-significant. Source data are provided as a Source Data file.

Supplementary Figure 8

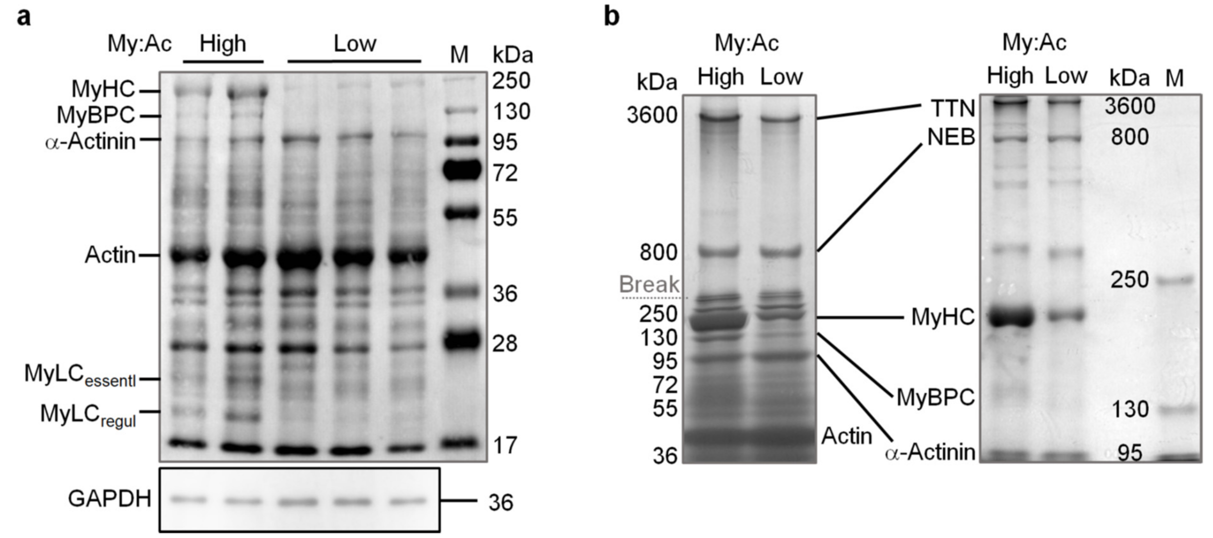

Supplementary Figure 8: Additional coomassie-stained gels of CIM patient muscles (m. tibialis anterior). a) 12.5% SDS-polyacrylamide gel loaded with human muscles expressing high or low myosin heavy chain (MyHC) over actin (My:Ac) ratios. The bottom panel shows a western blot using anti-GAPDH antibody, to demonstrate the similar loading. **b)** The left panel shows a titin gel (1.8% polyacrylamide) stacked onto a 6% running gel and loaded with biopsy samples of CIM muscles expressing a high or a low My:Ac ratio. The right panel shows a 2.8% polyacrylamide titin gel, which resolves proteins the size of ~4 MDa down to ~90 kDa. M, size marker. The gels shown are representative of a minimum of five similar gels per condition performed (for a given polyacrylamide concentration). Source data are provided as a Source Data file.

Supplementary Figure 9

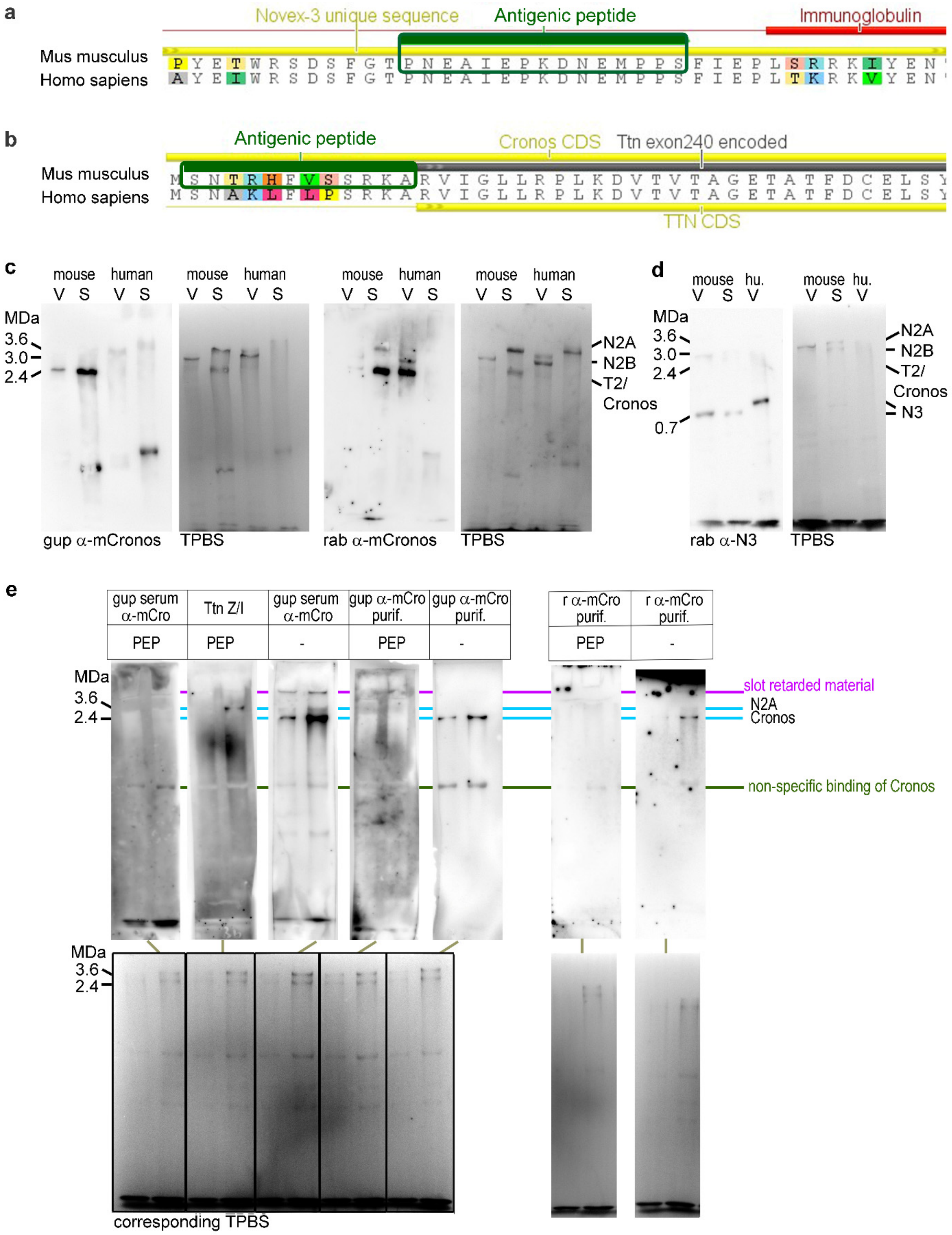

Supplementary Fig. 9: Validation of custom-made antibodies. a) and **b)** Green boxes indicate peptide sequences used for immunization of rabbits and guinea pigs to produce TTN isoform-specific antibodies against Novex-3 (a) and Cronos (b). For Novex-3 antibody production, a peptide sequence with 100% homology between mouse and human peptide sequences was chosen; in contrast, the murine Cronos sequence selected as the antigen has only 58% homology with the human Cronos sequence and two different antibodies are likely required. **c)** Cronos antibody cross-species reactivity test. Western blots of mouse and human muscle cell lysates from cardiac ventricle (V) and skeletal muscle (S) probed with the custom-made anti-mouse Cronos antibodies made either in

guinea pigs (gup α-mCronos) or in rabbits (rab α-mCronos). The total protein blot stain (TPBS) served as loading control and size standard. The mCronos antibodies detected the murine but not the human TTN Cronos protein. **d)** Novex-3 antibody cross-species reactivity test. Western blots of mouse and human muscle cell lysates from cardiac ventricle (V) and skeletal muscle (S) probed with the custom-made anti-Novex-3 antibody made in rabbits (rab α-N3). The total protein blot stain (TPBS) served as loading control and size standard. The rab α-N3 antibody detected the murine and the human TTN Novex-3 protein. **e)** Western blots from the peptide competition antibody specificity test. Blotted skeletal muscle cell lysates probed with serum from immunized animals or affinity purified (purif.) antibodies, which were either pre-blocked with the Cronos immunogenic peptide (PEP) or not. To rule out unspecific blocking by the peptide, one blot was incubated with the TTN Z/I antibody, which was pre-incubated with the Cronos immunogenic peptide. r α-mCro, rabbit anti-mouse Cronos antibody; gup α-mCro, guinea pig anti-mouse Cronos antibody. The total protein blot stain (TPBS) served as loading control. Gels/blots shown in panels c)-e) are representative of 2 gels/blots performed per condition. Source data are provided as a Source Data file.

Supplementary references:

1. Gramlich, M. *et al.* Stress-induced dilated cardiomyopathy in a knock-in mouse model mimicking human titin-based disease. *J Mol Cell Cardiol* **47**, 352-358 (2009).
2. Schafer, S. *et al.* Titin-truncating variants affect heart function in disease cohorts and the general population. *Nat Genet* **49**, 46-53 (2017).
3. Radke, M.H. *et al.* Deleting full length titin versus the titin M-band region leads to differential mechanosignaling and cardiac phenotypes. *Circulation* **139**, 1813-1827 (2019).
4. Kramer, A.W., Jr. & Marks, L.S. The occurrence of cardiac muscle in the pulmonary veins of Rodenita. *J Morphol* **117**, 135-149 (1965).
