# Supplementary material for: Maintenance of sarcomeric integrity in adult muscle cells crucially depends on Z-disc anchored titin
Source: Nat Commun. 2020 Sep 8;11:4479. doi: 10.1038/s41467-020-18131-2 (PMC7478974; doi:10.1038/s41467-020-18131-2)
Supplement: Supplementary file 3 — Reporting Summary [file 41467_2020_18131_MOESM3_ESM.pdf]

nature research

Corresponding author(s): Wolfgang A. Linke, Sandra Swist

Last updated by author(s): July 03, 2020

Reporting Summary

Nature Research wishes to improve the reproducibility of the work that we publish. This form provides structure for consistency and transparency in reporting. For further information on Nature Research policies, see Authors & Referees and the Editorial Policy Checklist .

Statistics

For all statistical analyses, confirm that the following items are present in the figure legend, table legend, main text, or Methods section.

n/a	Confirmed	
☐	☒	The exact sample size (*n*) for each experimental group/condition, given as a discrete number and unit of measurement
☐	☒	A statement on whether measurements were taken from distinct samples or whether the same sample was measured repeatedly
☐	☒	The statistical test(s) used AND whether they are one- or two-sided<br>*Only common tests should be described solely by name; describe more complex techniques in the Methods section.*
☒	☐	A description of all covariates tested
☐	☒	A description of any assumptions or corrections, such as tests of normality and adjustment for multiple comparisons
☐	☒	A full description of the statistical parameters including central tendency (e.g. means) or other basic estimates (e.g. regression coefficient) AND variation (e.g. standard deviation) or associated estimates of uncertainty (e.g. confidence intervals)
☒	☐	For null hypothesis testing, the test statistic (e.g. *F*, *t*, *r*) with confidence intervals, effect sizes, degrees of freedom and *P* value noted<br>*Give P values as exact values whenever suitable.*
☒	☐	For Bayesian analysis, information on the choice of priors and Markov chain Monte Carlo settings
☒	☐	For hierarchical and complex designs, identification of the appropriate level for tests and full reporting of outcomes
☒	☐	Estimates of effect sizes (e.g. Cohen's *d*, Pearson's *r*), indicating how they were calculated

Our web collection on statistics for biologists contains articles on many of the points above.

Software and code

Policy information about availability of computer code

Data collection	Chemiluminescence on western blots and Coomassie stained gels were recorded using the Image Quant software v7.1 of the ImageQuant LAS 4000 Imaging System (GE Healthcare). Stained tissue sections were imaged with NIS Elements Software v4.3 provided with the Nikon DS-Fi2/DS-U3 camera. Electron microscopic images were taken with a TRS sharpeye CCD Camera and manufacturer's software ImageSP (Troendle, Moorenweis, Germany).<br>Immunofluorescence images from tissue sections were recorded using NIS Elements software v.3.1 as part of a confocal laser scanning system (Nikon A1). qPCR data were collected by the StepOne™ Real-Time PCR System and StepOne Software v2.3 (Applied Biosystems). AFM nanoindentation was done using the Nanowizard 3 AFM system and JPK data processing software v4.2 (JPK systems, Berlin, Germany). Passive and active tension measurements of myofibers were performed using the manufacturer's software on a custom-made setup by Scientific Instruments, Heidelberg (Germany).
Data analysis	Chemiluminescence signal intensity was quantified using the ImageQuant TL software v7.1 (GE Healthcare).<br>Digital images of hematoxylin/eosin-stained tissue sections were analyzed by the ImageJ software v1.51j8 (NIH).<br>Data organization, scientific graphing and statistical analyses were performed using Microsoft Excel (2013) and GraphPad Prism v7.04.<br>Analysis of Z-disc circumference was done using ImageSP, TRS Systems, Moorenweis, Germany.

For manuscripts utilizing custom algorithms or software that are central to the research but not yet described in published literature, software must be made available to editors/reviewers. We strongly encourage code deposition in a community repository (e.g. GitHub). See the Nature Research guidelines for submitting code & software for further information.

Data

Policy information about availability of data

All manuscripts must include a data availability statement. This statement should provide the following information, where applicable:

- Accession codes, unique identifiers, or web links for publicly available datasets
- A list of figures that have associated raw data
- A description of any restrictions on data availability

All data pertaining to this work are shown in the text, figures, and Supplementary Information. The source data underlying Figs. 1e, 2a, b, d, e, 3c, g, 4b-d, 5a-d, 6b, d, 7b-d, 8a, d, and supplementary figures 2e, 5b, 6b-d, and 7 are provided as a Source Data file. Full scans of images shown in panels 1c, d; 5b, d; 6a, c; 7a, c, d; 8b, c, d, and supplementary figures 2b, d; 6c; 8a, b, and 9e are also provided as a separate Source Data file.

Field-specific reporting

Please select the one below that is the best fit for your research. If you are not sure, read the appropriate sections before making your selection.

☒ Life sciences ☐ Behavioural & social sciences ☐ Ecological, evolutionary & environmental sciences

For a reference copy of the document with all sections, see nature.com/documents/nr-reporting-summary-flat.pdf

Life sciences study design

All studies must disclose on these points even when the disclosure is negative.

Sample size	No specific sample size calculation was undertaken before experiments. We chose sample size according to standard practice in the field of muscle physiology, which suggested that in most cases, n=5-7 animals/group are sufficient for characterization of differences in genotype, phenotype, and specific heart or muscle properties (e.g., DOI: 10.1161/CIRCULATIONAHA.106.645499 and DOI: 10.1126/science.aaa5458).
Data exclusions	No data were excluded.
Replication	For qPCRs analyses, technical duplicates were used to control for the validity of the method. The in vivo experiments on adult mice were performed on 7-11 individual animals/group. Post mortem tissue analyses were performed on tissues from at least 2 individual mice/group, except the lacZ stainings in the reporter line R26R/ACTA1-rtTA;tetO-cre; here, only one animal was analyzed to test for the effectiveness of the doxycycline treatment. Otherwise, the number of in vitro experiments and number of biologically independent samples are mentioned in the figure legends. All data shown could be reproduced in multiple repeats, as indicated in the figure legends.
Randomization	Experiments were not randomized. For the mouse studies, littermates were grouped by genotypes (WT vs MUT) and both groups treated with doxycycline in parallel. As there were no further treatment groups, there was no additional randomization.
Blinding	Investigators were not blinded. Blinded analysis was not done, because in most experiments genotypes were immediately recognized by phenotypic differences, e.g., pronounced weight loss in MUT vs. WT mice. Likewise, protein lysates from WT and MUT were readily distinguished by total protein stain and microscopical images by ultrastructure. These obvious differences hindered blinding and made it less relevant.

Reporting for specific materials, systems and methods

We require information from authors about some types of materials, experimental systems and methods used in many studies. Here, indicate whether each material, system or method listed is relevant to your study. If you are not sure if a list item applies to your research, read the appropriate section before selecting a response.

Materials & experimental systems

n/a	Involved in the study	
☐	☒	Antibodies
☒	☐	Eukaryotic cell lines
☒	☐	Palaeontology
☐	☒	Animals and other organisms
☐	☒	Human research participants
☒	☐	Clinical data

Methods

n/a	Involved in the study	
☒	☐	ChIP-seq
☒	☐	Flow cytometry
☒	☐	MRI-based neuroimaging

Antibodies

Antibodies used	A complete list containing full information about the antibodies is provided in Supplementary Table 2. For all antibodies, we have provided the supplier name, catalog number, and validation method.
Validation	Validation statements for the primary antibodies purchased from a manufacturer are provided in Supplementary Table 2, as obtained from their websites, specifying for species and application type. For custom-made antibodies validated in this study, the following validation statements are provided:<br>-TTN gup-anti-mouse Cronos: Validated in this study by WB & IHC. In addition to Cronos, the antibody detects an unspecific band of ~0.75 MDa).<br>-TTN MIR: Validated by IEM in WT and knockout mice in the present study.<br>-TTN Novex-3: Validated by WB and IHC of WT and knockout mice in the present study.<br>-TTN rabbit-anti-mouse Cronos: Validated in this study by WB of WT and knockout muscle (in addition to Cronos, the antibody detects an unspecific band of ~0.75 MDa).

Animals and other organisms

Policy information about studies involving animals; ARRIVE guidelines recommended for reporting animal research

Laboratory animals	Mus musculus of background strain C57BL/6JRj were used, age 8-45 weeks, both male and female. Genetic modifications are as indicated in the paper. For generation of chimeric titin knockout-first mice, blastocysts of wildtype BAlb/C mice were used. Mice were housed in open cages, closed with a mesh lid. Quarterly health monitoring was carried out according to Felasa regulations. For custom-made antibody production, the authorized company immunized rabbits and guinea pigs.
Wild animals	none
Field-collected samples	none
Ethics oversight	All animal procedures were approved by local governmental authorities (Landesamt für Natur, Umwelt und Verbraucherschutz Nordrhein-Westfalen, LAN UV, Az 84-02.04.2015.A261).

Note that full information on the approval of the study protocol must also be provided in the manuscript.

Human research participants

Policy information about studies involving human research participants

Population characteristics	Intensive care unit (ICU) patients with critical illness myopathy (CIM) (intermediate myosin:actin ratios: 3 warnen and 2 men, 62-79 years; low myosin:actin ratios: 3 warnen and 1 man, 41-79 years) and 5 control immobilized ICU patients with lesions in the central or peripheral nervous system (2 warnen and 3 men, 56-70 years). All lCU patients with CIM had been mechanically ventilated for langer than two weeks at the time of muscle biopsy.
Recruitment	The biopsies are from patients recruited in a study aiming at improving the diagnostic precision of CIM, i.e., comparing results from percutaneous muscle biopsies with results from biopsies obtained with a disposable microbiopsy instrument. Thus, ICU patients were included who had developed a general paralysis in response to long-term mechanical ventilation and were referred to the clinic for diagnostic purposes. Control samples displaying high myosin:actin ratios were obtained on the first day on the ventilator prior to any myosin loss. In these patients, a myosin loss was evident first after more than one week of immobilization and mechanical ventilation. There is no indication that the inclusion of these patients may have introduced any bias which may have impacted on the results.
Ethics oversight	The study was approved by the Ethical Committee an Human Research at Karolinska lnstitutet, Stockholm and Uppsala University Hospital, Uppsala, Sweden.

Note that full information on the approval of the study protocol must also be provided in the manuscript.
