# Supplementary material for: Maintenance of sarcomeric integrity in adult muscle cells crucially depends on Z-disc anchored titin
Source: Nat Commun. 2020 Sep 8;11:4479. doi: 10.1038/s41467-020-18131-2 (PMC7478974; doi:10.1038/s41467-020-18131-2)

Figure 1c

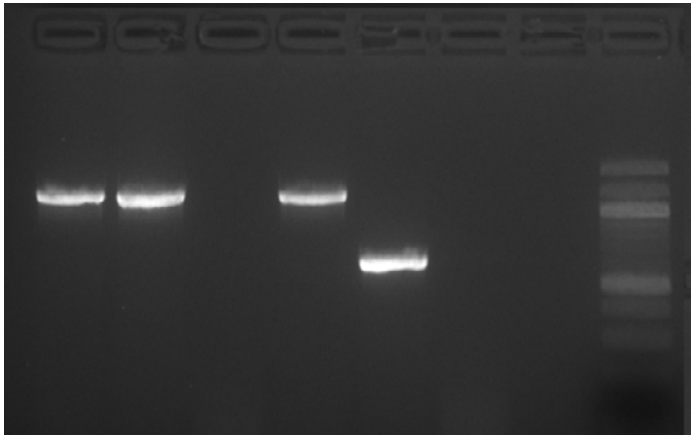

Ttn exon2-exon8 PCR; Marker: 100 bp ladder, NEB, N3231S

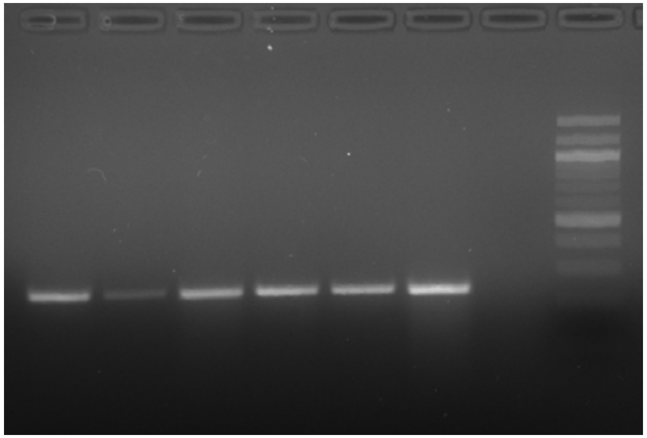

Hprt PCR; Marker: 100 bp ladder, NEB, N3231S

Figure 1d

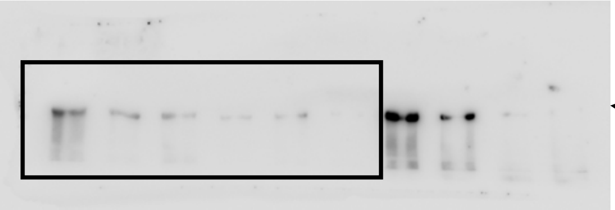

WB: Anti-Ttn Z/I (2080)

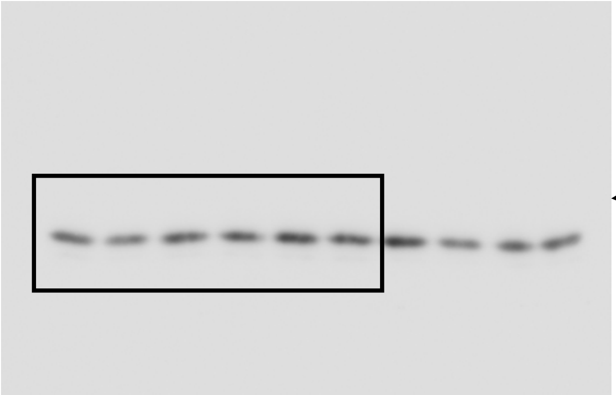

WB: Anti-GAPDH;

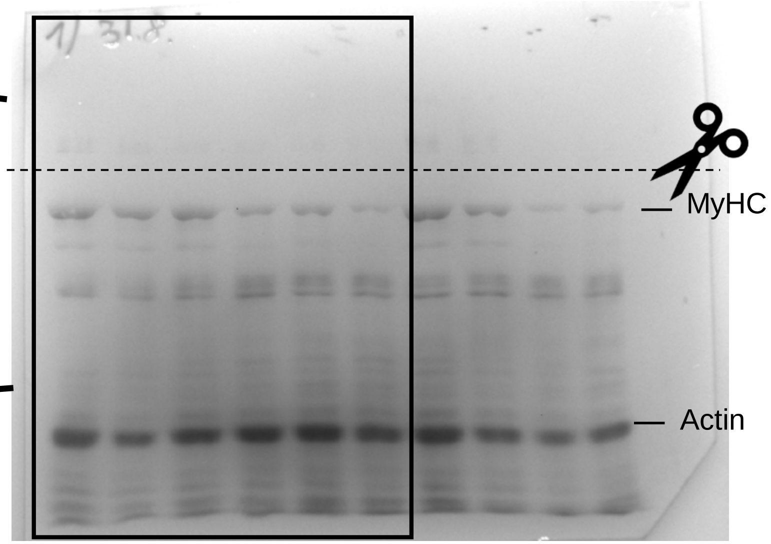

Corresponding total protein blot stain.
Western blot of 2% Ttn gel stacked on 8% polyacrylamide gel.

Figure 5b

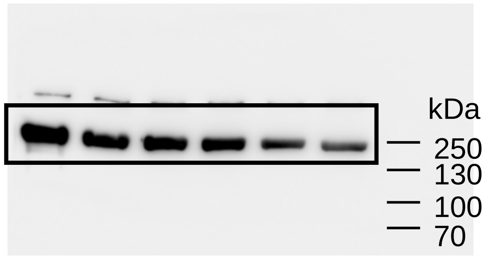

Western blot, of 10% SDS page,
Upper half incubated with **anti-Myh4**

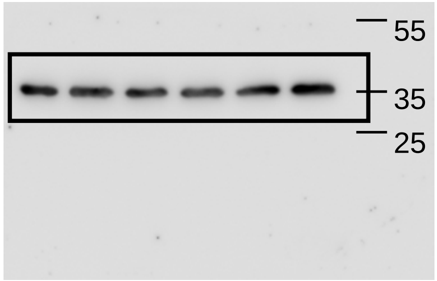

Western blot, of 10% SDS page,
Lower half incubated with **anti-GAPDH**

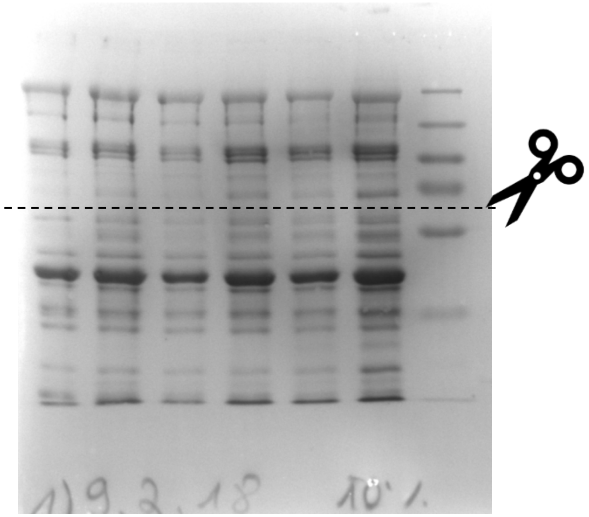

Corresponding total protein blot stain.

Figure 5d

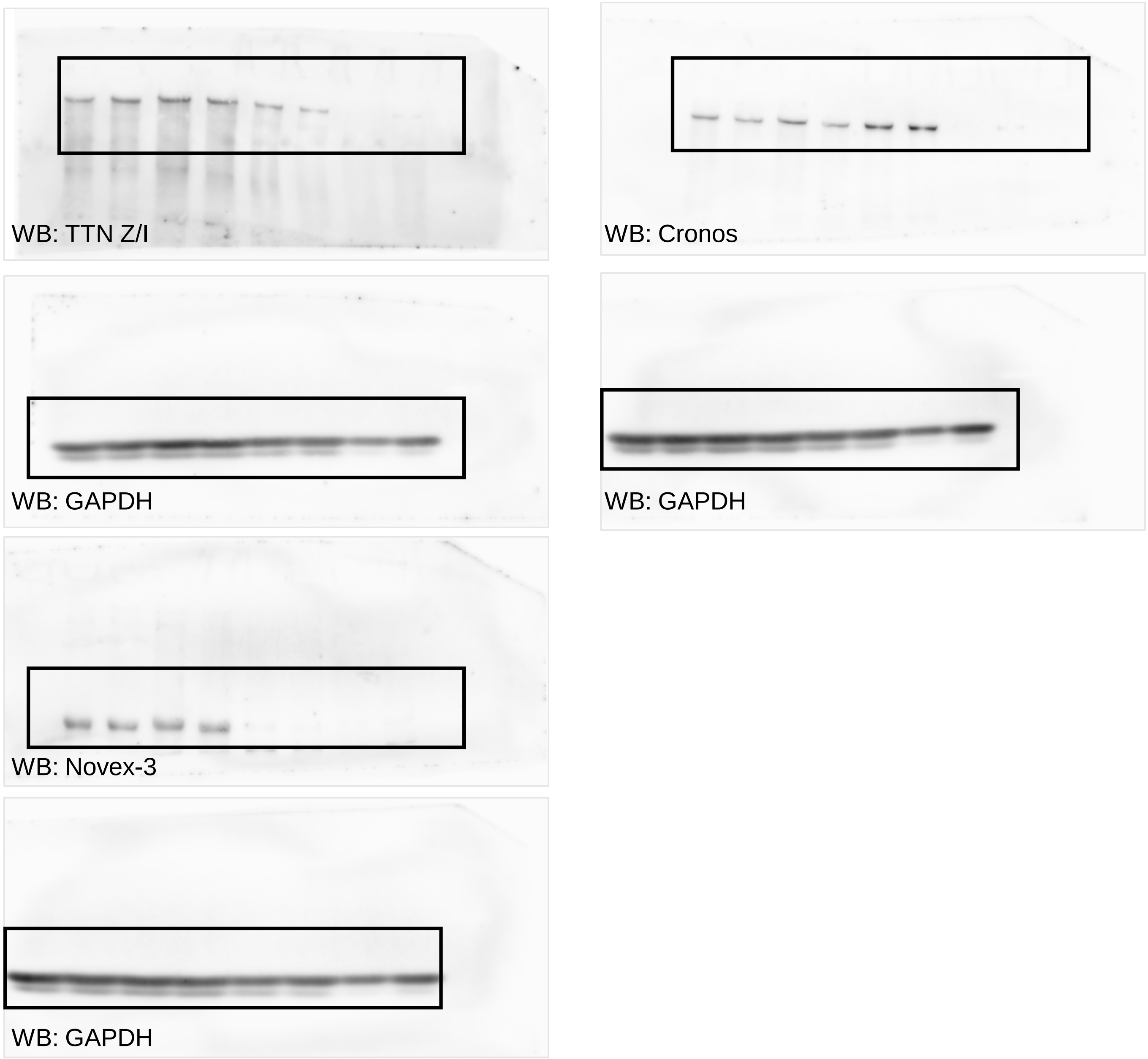

Figure 6a

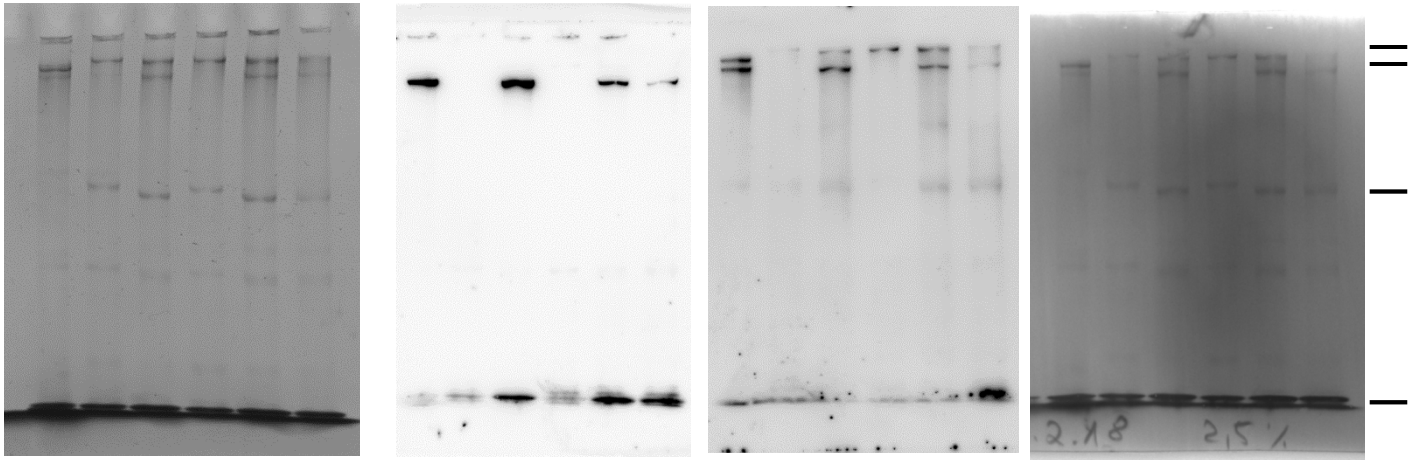

2.5% TTN gel, Coomassie | WB: Cronos | WB: Cronos + TTN Z/I | WB: TPBS

Figure 6c Blotting of TTN gels stacked onto 10% polyacrylamid gels. The blots were cut before the incubation with the antibodies.

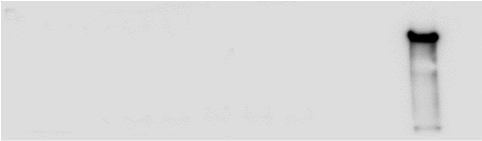

WB: 1st incubation with anti-Cronos

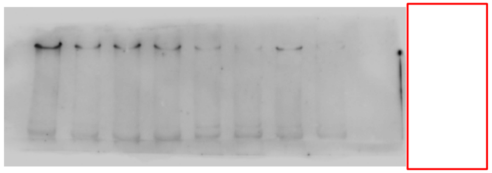

WB: 2nd incubation with TTN Z/I (N2A)

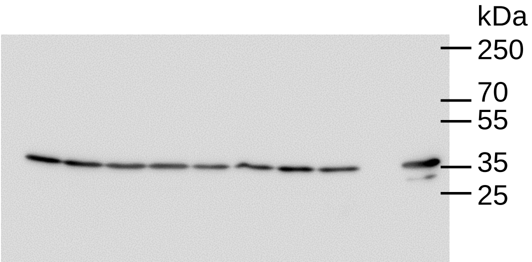

WB: GAPDH

BEFORE IMAGING THIS PART OF THE MEMBRANE WAS REMOVED; BCAUSE THE STRONG SIGNAL OF THE POSITIVE CONTROL HINDEREs VISUALIZATION OF THE N2A SIGNAL.

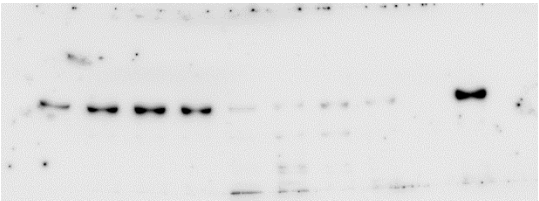

WB: Novex-3

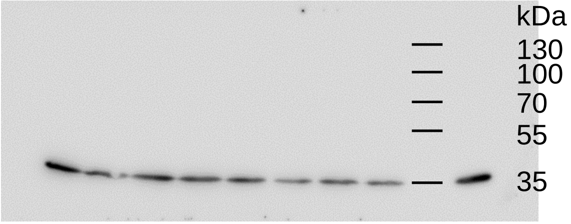

WB: GAPDH

Figure 7a

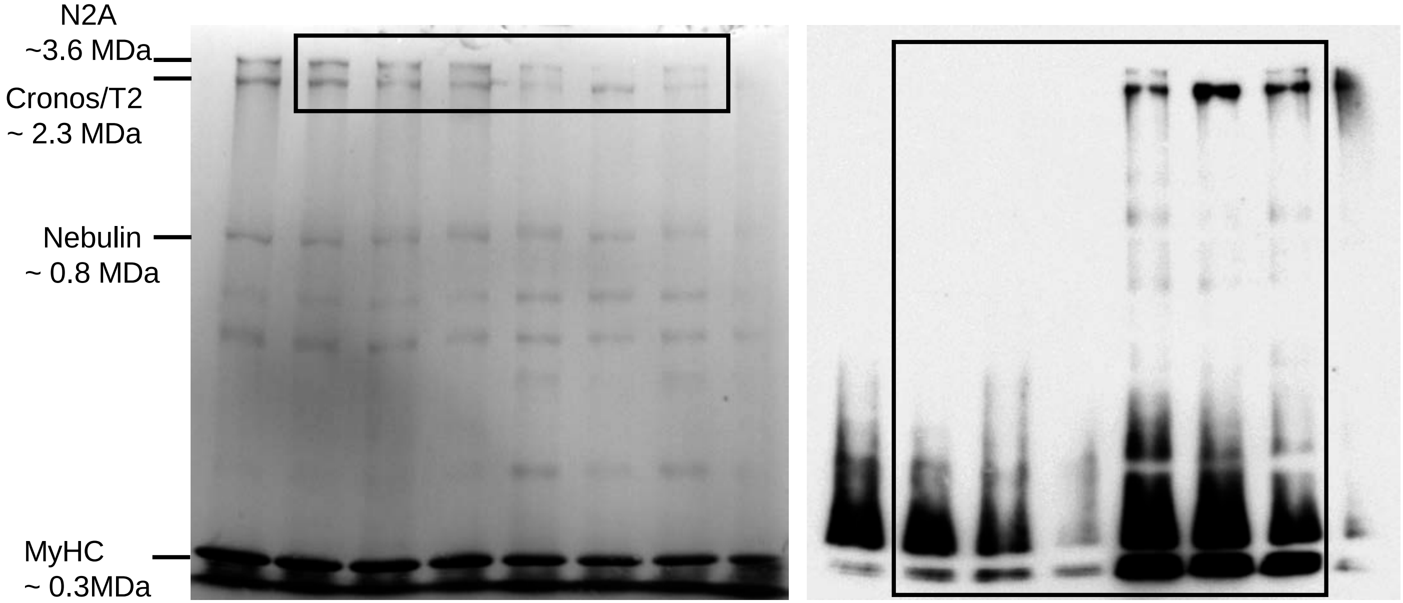

WB: Total Protein Blot Stain, WB of 2.5% gel

WB: anti-Ubiquitin

Figure 7c

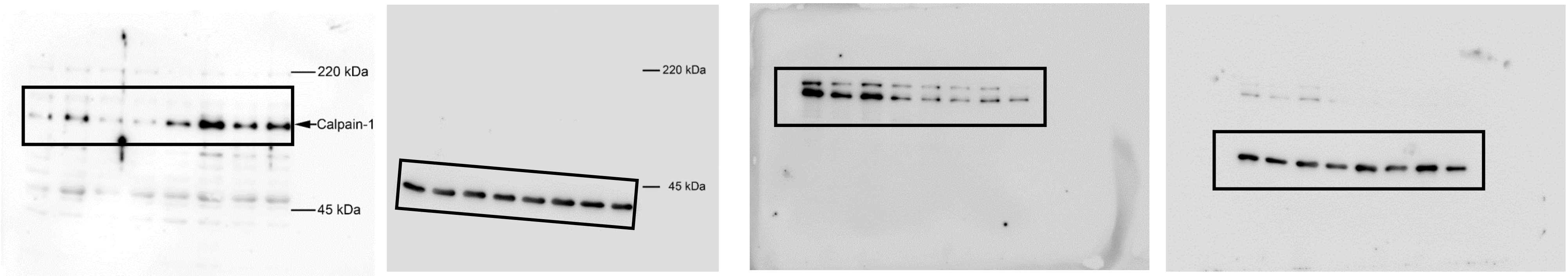

WB: CAPN1

WB: ~~CAPN1~~, GAPDH

WB: CAPN3

WB: ~~CAPN3~~, GAPDH

Figure 7c

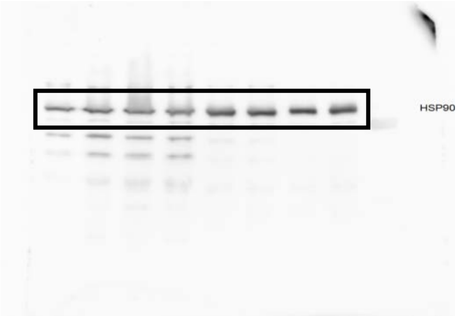

WB: HSP90

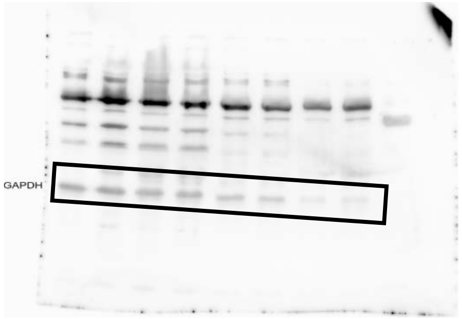

WB: ~~HSP90~~, GAPDH

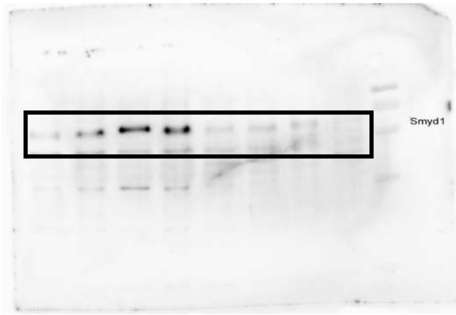

WB: SMYD1

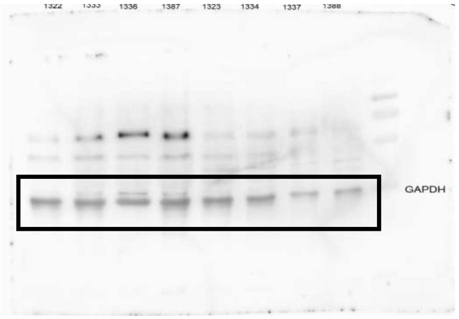

WB: ~~SMYD1~~, GAPDH

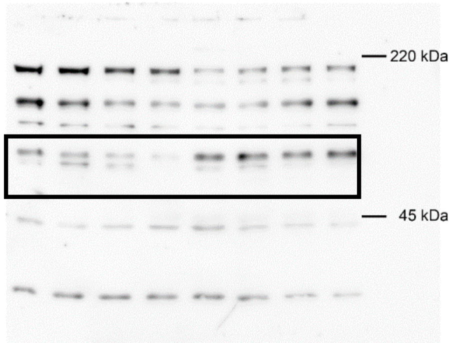

WB: SQSTM1

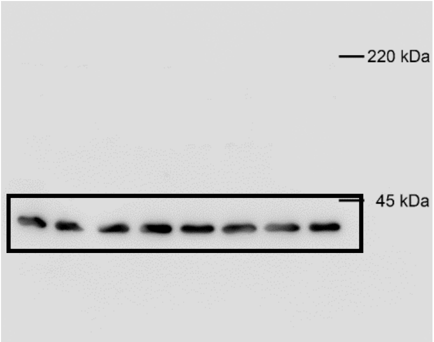

WB:~~SQSTM1~~, GAPDH

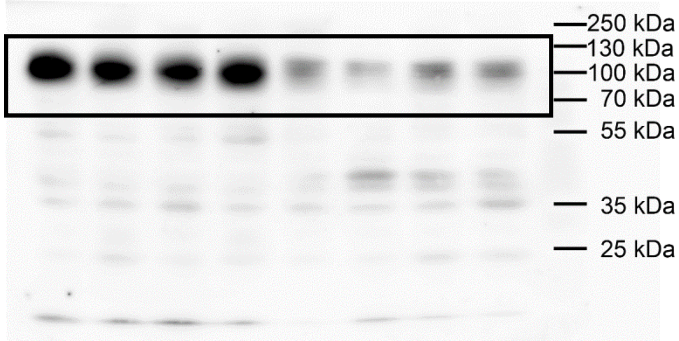

WB: LAMP2

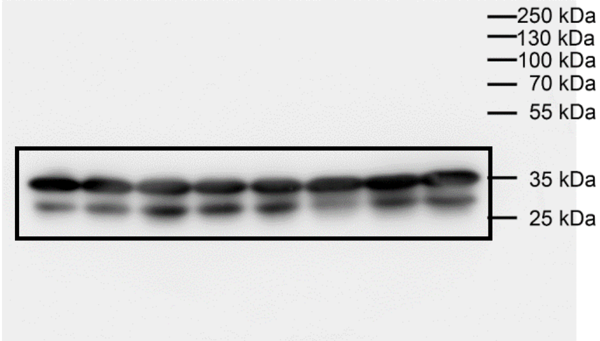

WB: ~~LAMP2~~, GAPDH

Figure 7d

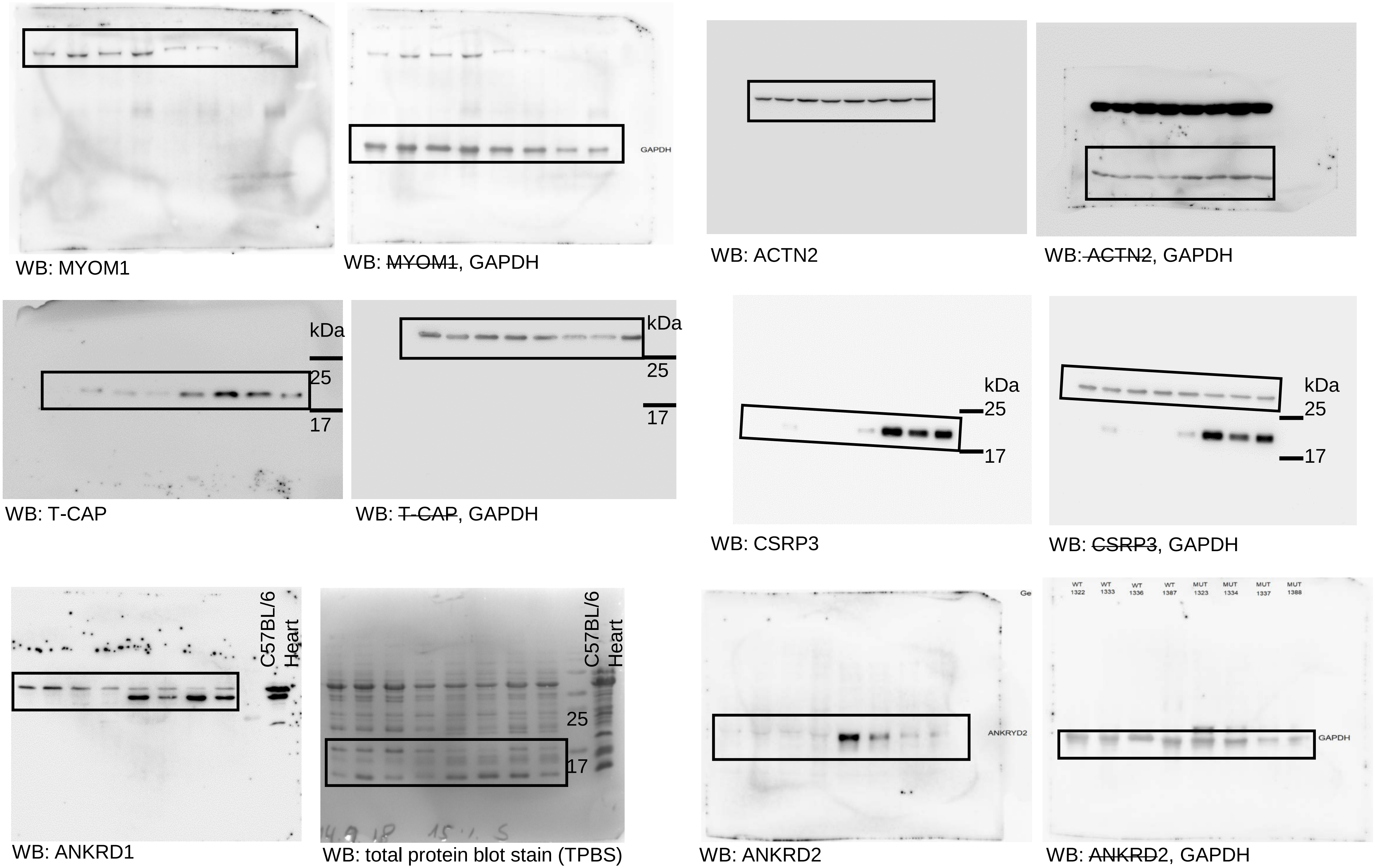
WB: MYOM1
GAPDH
WB: ~~MYOM1~~, GAPDH
WB: ACTN2
WB: ~~ACTN2~~, GAPDH
kDa
25
17
WB: T-CAP
kDa
25
17
WB: ~~T-CAP~~, GAPDH
kDa
25
17
WB: CSRP3
kDa
25
17
WB: ~~CSRP3~~, GAPDH
C57BL/6
Heart
WB: ANKRD1
C57BL/6
Heart
25
17
WB: total protein blot stain (TPBS)
ANKRYD2
WB: ANKRD2
WT 1322
WT 1333
WT 1336
WT 1387
MUT 1323
MUT 1334
MUT 1337
MUT 1388
GAPDH
WB: ~~ANKRD~~2, GAPDH

Figure 8b

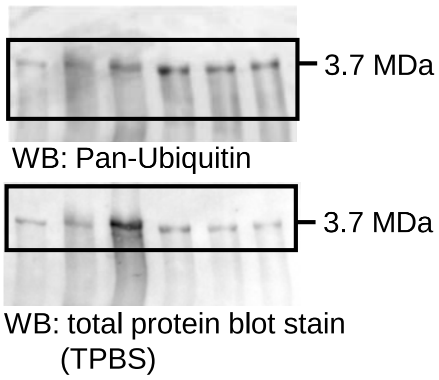

Figure 8c

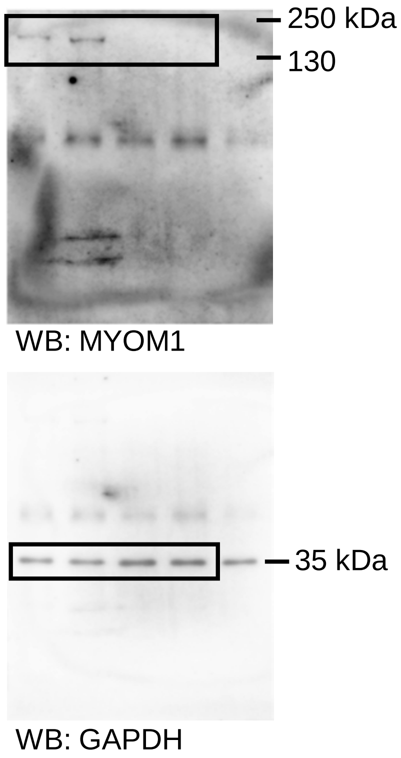

Figure 8d

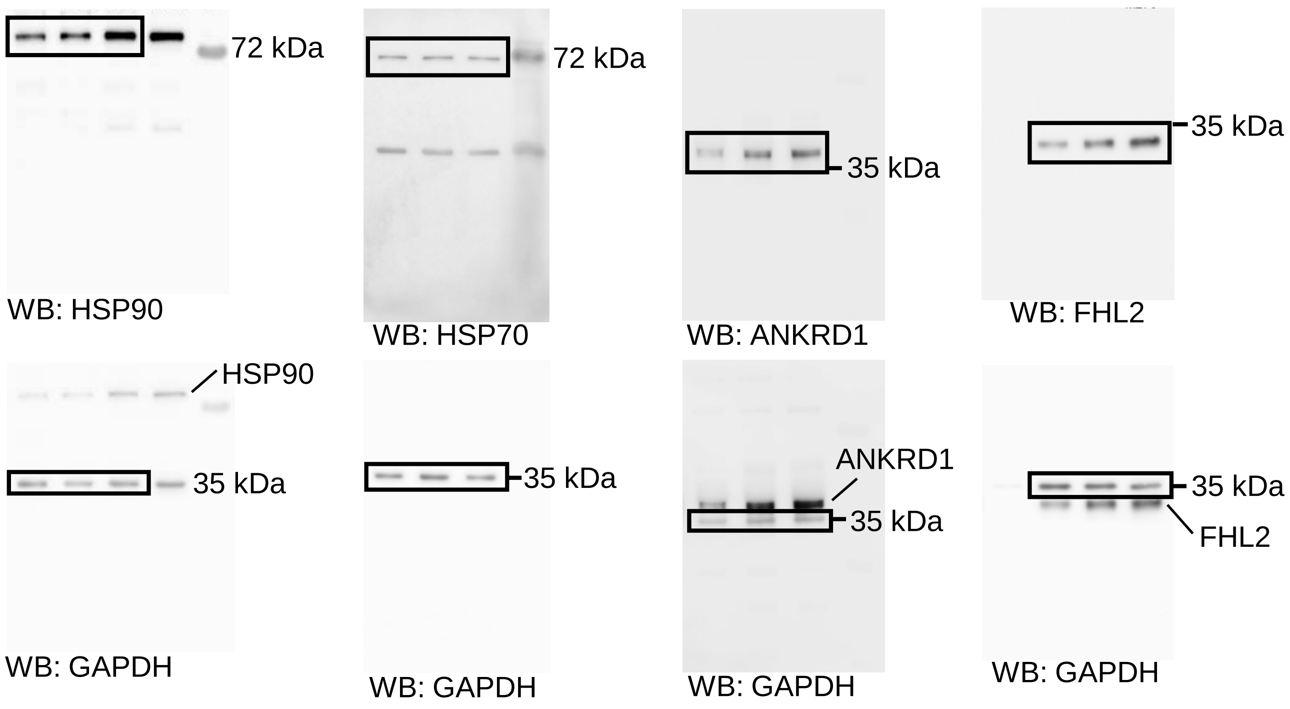

Supplementary Figure 2b

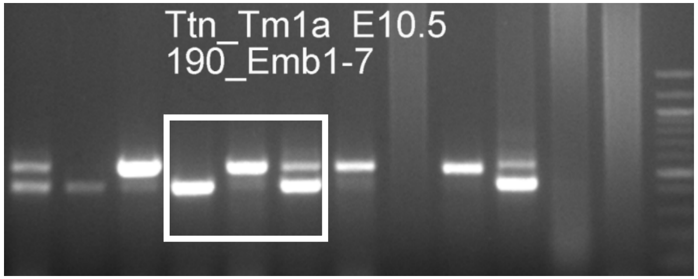

PCR: *Titin* genotyping with primers P1, P3, P4)

Supplementary Figure 2d

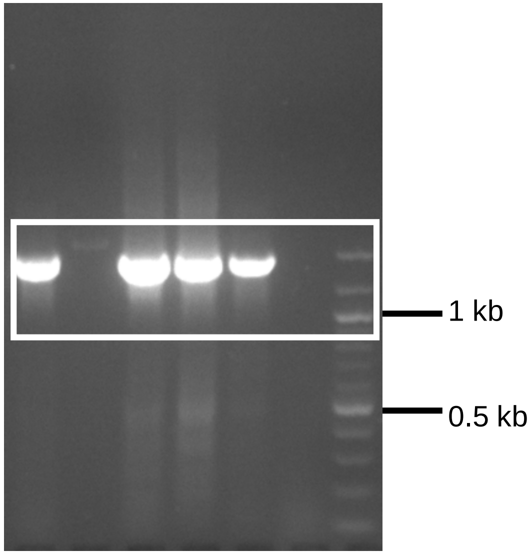

PCR: Ttn exon 1-8

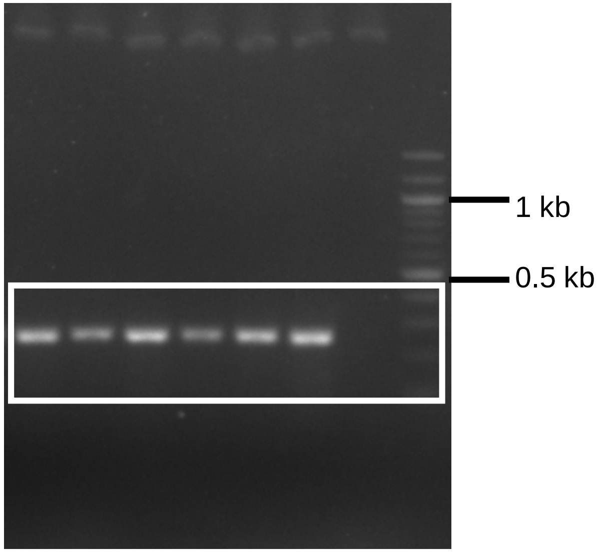

PCR: Hprt

Supplementary Figure 6 b

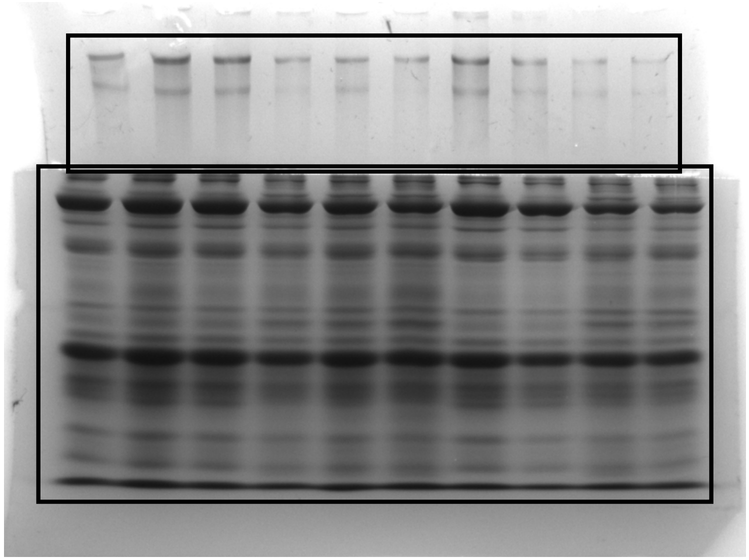

TTN gel stacked on polyacrylamide gel, Commassie stained

Supplementary Figure 6 c – Middle panel

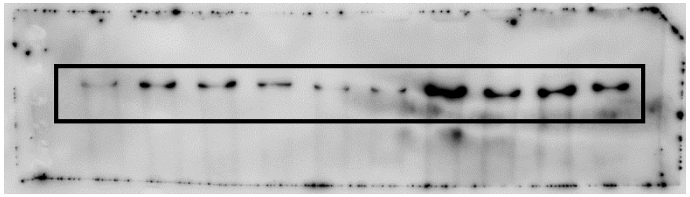

WB: Cronos

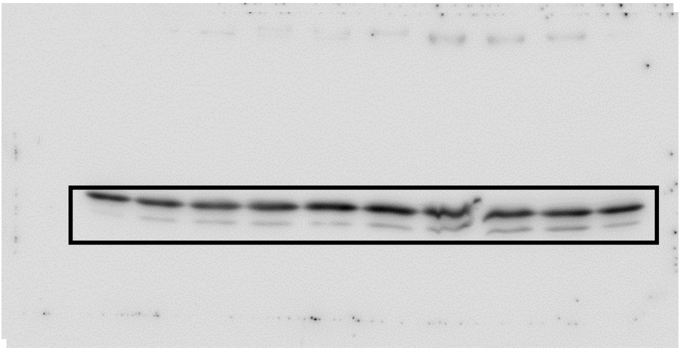

WB: GAPDH

Supplementary Figure 6 c – Upper panel

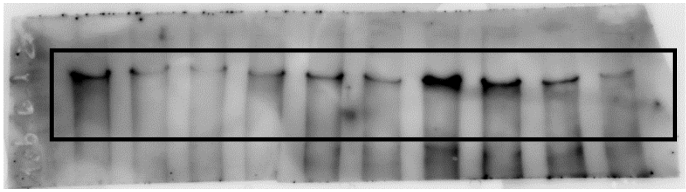

WB: TTN Z/I (2080)

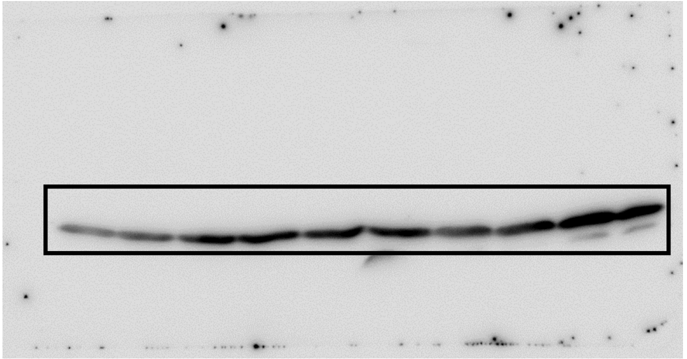

WB: GAPDH

Supplementary Figure 6 c – Lower panel

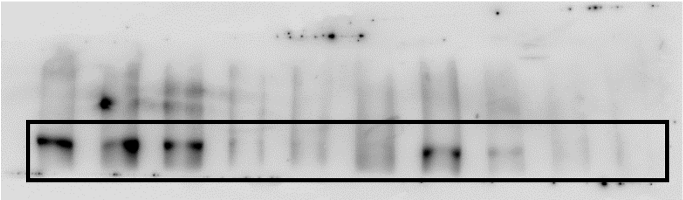

WB: Novex-3

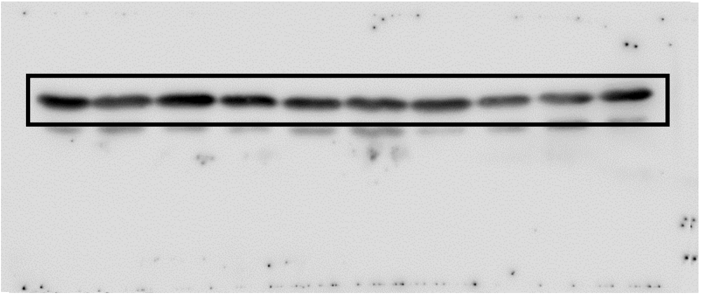

WB: GAPDH

Supplementary Figure 8a

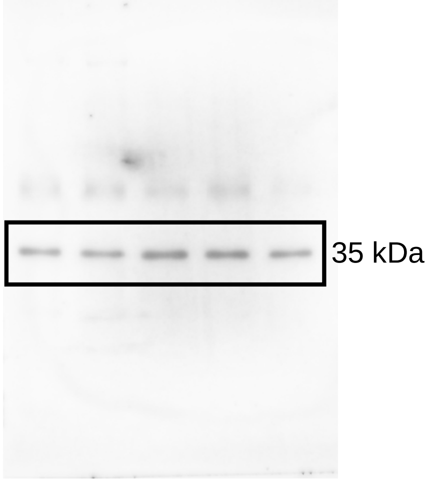

35 kDa

WB: GAPDH

Supplementary Figure 8b, left

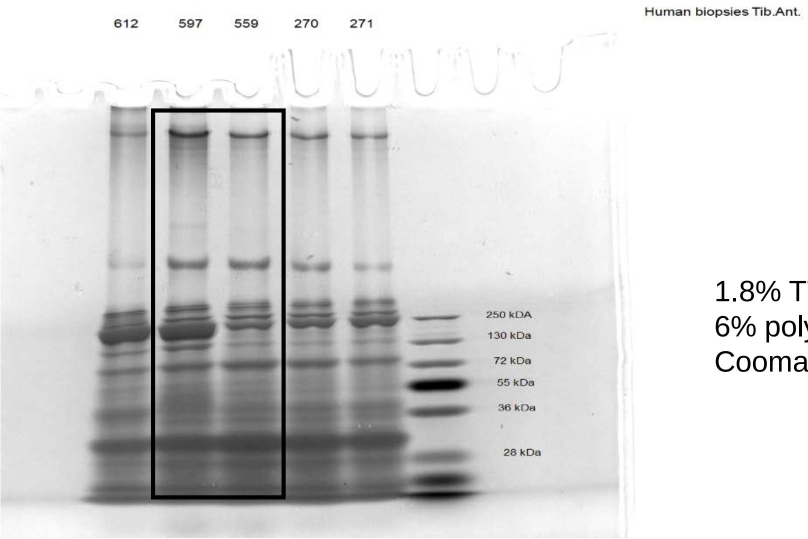

1.8% TTN gel, stacked on 6% polyacrylamide gel, Coomassie

Supplementary Figure 8b, right

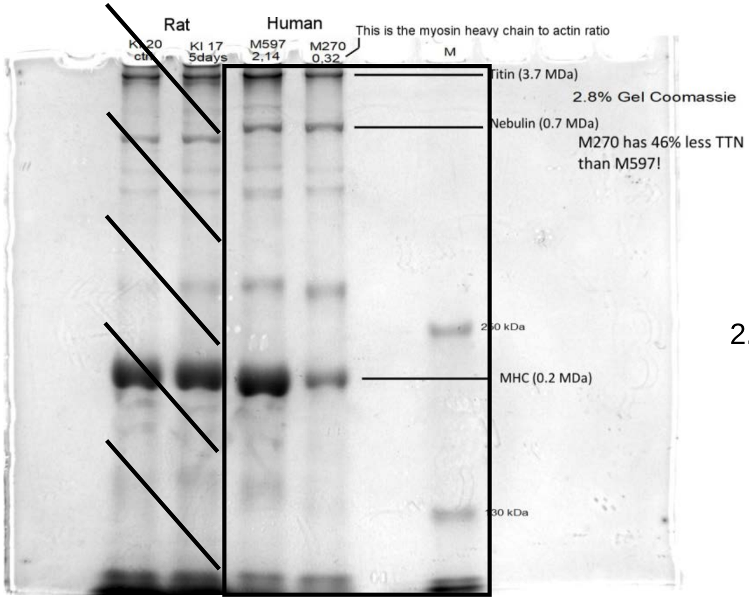

2.8% TTN gel, Coomassie

Supplementary Figure 9c

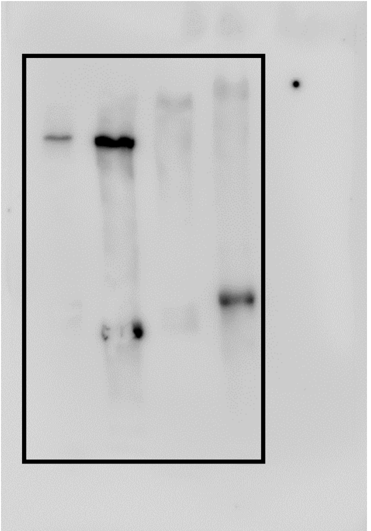

WB: gup-α-mCronos

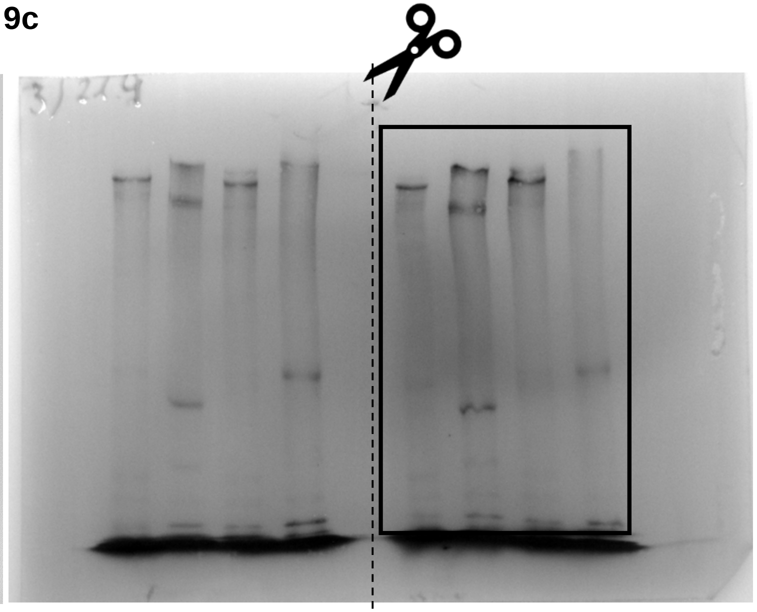

WB: total protein blot stain (TPBS)

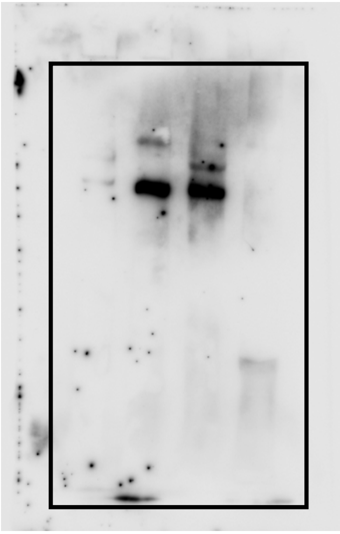

WB: rab-α-mCronos

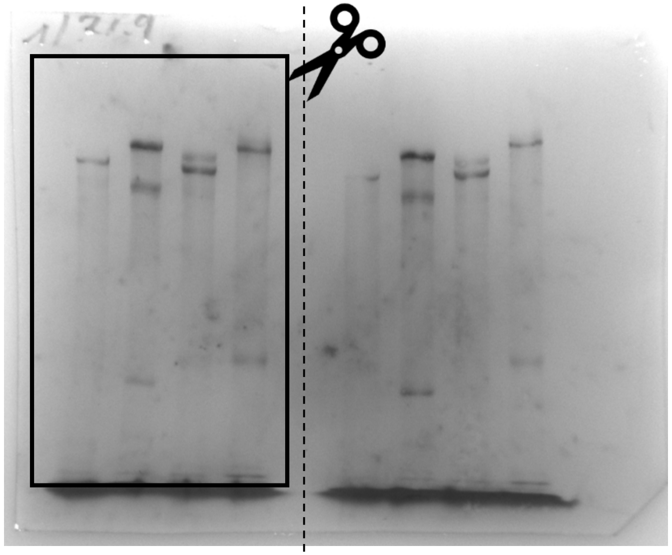

WB: total protein blot stain (TPBS)

Supplementary Figure 9d

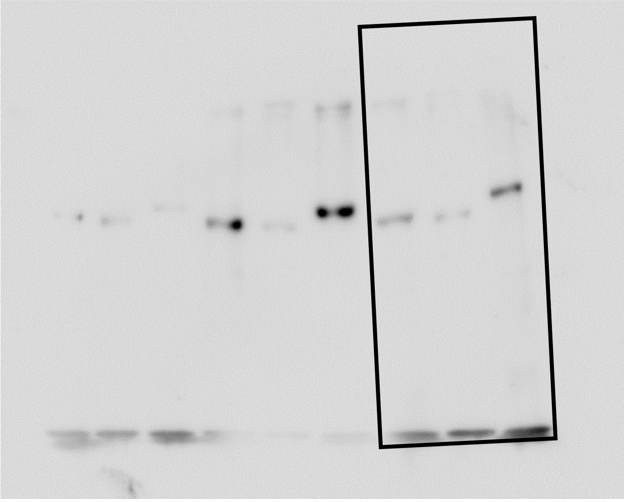

WB: rab-α-N3

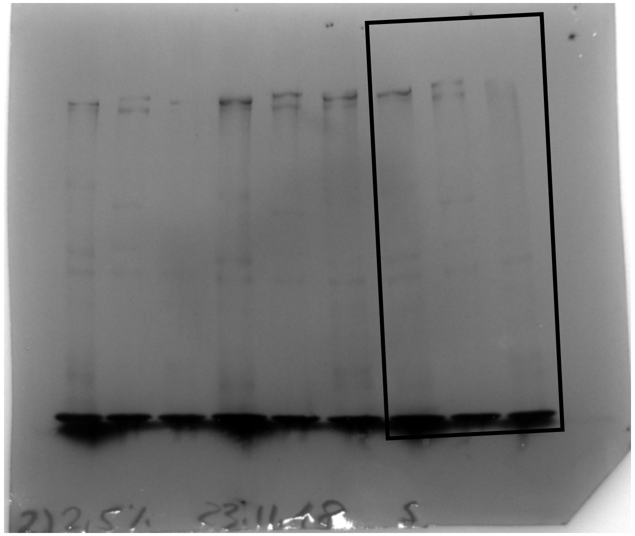

WB: total protein blot stain (TPBS)

Supplementary Figure 9e

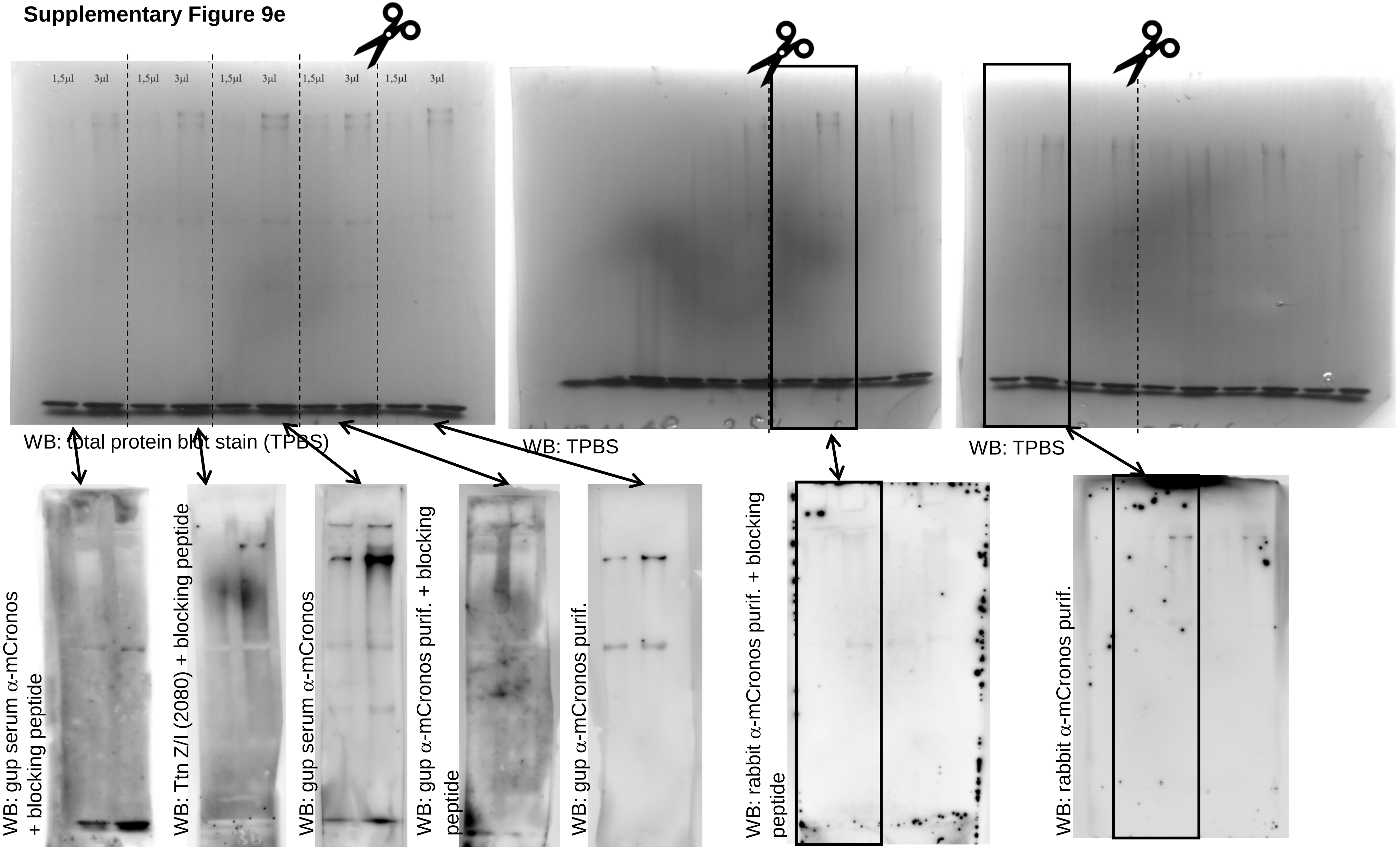

Supplement: Supplementary file 4 — Source Data [file 41467_2020_18131_MOESM4_ESM.zip › Source data Blots_Revision_Final.pdf]
